# Selection signatures in two oldest Russian native cattle breeds revealed using high-density single nucleotide polymorphism analysis

**DOI:** 10.1371/journal.pone.0242200

**Published:** 2020-11-16

**Authors:** Natalia Anatolievna Zinovieva, Arsen Vladimirovich Dotsev, Alexander Alexandrovich Sermyagin, Tatiana Evgenievna Deniskova, Alexandra Sergeevna Abdelmanova, Veronika Ruslanovna Kharzinova, Johann Sölkner, Henry Reyer, Klaus Wimmers, Gottfried Brem

**Affiliations:** 1 L.K. Ernst Federal Science Center for Animal Husbandry, Federal Agency of Scientific Organizations, settl. Dubrovitzy, Podolsk Region, Moscow Province, Russia; 2 Division of Livestock Sciences, University of Natural Resources and Life Sciences, Vienna, Austria; 3 Institute of Genome Biology, Leibniz Institute for Farm Animal Biology [FBN], Dummerstorf, Germany; 4 Institute of Animal Breeding and Genetics, University of Veterinary Medicine [VMU], Vienna, Austria; Banaras Hindu University, INDIA

## Abstract

Native cattle breeds can carry specific signatures of selection reflecting their adaptation to the local environmental conditions and response to the breeding strategy used. In this study, we comprehensively analysed high-density single nucleotide polymorphism (SNP) genotypes to characterise the population structure and detect the selection signatures in Russian native Yaroslavl and Kholmogor dairy cattle breeds, which have been little influenced by introgression with transboundary breeds. Fifty-six samples of pedigree-recorded purebred animals, originating from different breeding farms and representing different sire lines, of the two studied breeds were genotyped using a genome-wide bovine genotyping array (Bovine HD BeadChip). Three statistical analyses—calculation of fixation index (*F*_ST_) for each SNP for the comparison of the pairs of breeds, hapFLK analysis, and estimation of the runs of homozygosity (ROH) islands shared in more than 50% of animals—were combined for detecting the selection signatures in the genome of the studied cattle breeds. We confirmed nine and six known regions under putative selection in the genomes of Yaroslavl and Kholmogor cattle, respectively; the flanking positions of most of these regions were elucidated. Only two of the selected regions (localised on BTA 14 at 24.4–25.1 Mbp and on BTA 16 at 42.5–43.5 Mb) overlapped in Yaroslavl, Kholmogor and Holstein breeds. In addition, we detected three novel selection sweeps in the genome of Yaroslavl (BTA 4 at 4.74–5.36 Mbp, BTA 15 at 17.80–18.77 Mbp, and BTA 17 at 45.59–45.61 Mbp) and Kholmogor breeds (BTA 12 at 82.40–81.69 Mbp, BTA 15 at 16.04–16.62 Mbp, and BTA 18 at 0.19–1.46 Mbp) by using at least two of the above-mentioned methods. We expanded the list of candidate genes associated with the selected genomic regions and performed their functional annotation. We discussed the possible involvement of the identified candidate genes in artificial selection in connection with the origin and development of the breeds. Our findings on the Yaroslavl and Kholmogor breeds obtained using high-density SNP genotyping and three different statistical methods allowed the detection of novel putative genomic regions and candidate genes that might be under selection. These results might be useful for the sustainable development and conservation of these two oldest Russian native cattle breeds.

## Introduction

National livestock genetic resources are the most important carriers of the unique forms of variability; they are necessary to ensure the sustainability of local agricultural production systems [1]. In Russia, over a long period, various cattle breeds have formed, which are characterised by good adaptation to the local environmental conditions and better suitability for the development of geographic production systems [2]. Among the Black-and-White cattle breeds, Kholmogor and Yaroslavl dairy cattle have remarkable importance for animal husbandry in the Central and Northern regions of European Russia. Both breeds originated from small-stature Great Russian Cattle (height at withers is 105–110 cm, body weight up to 325–390 kg [3, 4]. The first records of the presence of cattle on the islands in the upper reaches of the Northern Dvina River near the Kholmogory town in Northern Russia date back to the sixteenth century (the breed was named after this town) [5]. Over a long history, the good pastures and hayfields in the Kholmogor breeding zone have contributed to the development of the tall cattle breed well suited for dairy production.

In the 18th–19th centuries, the Kholmogor cattle were well-known in Russia and abroad owing to their high productivity and excellent quality of dairy products. Cattle breeding began to develop in the Yaroslavl region (near Moscow), based on which the breed is named, in the seventeenth and eighteenth centuries [5]. The Yaroslavl cattle were selected for adaptability, high feed efficiency and good reproductive ability even in poor forage conditions during winter. Moreover, this breed was in great demand among peasants and small cattle holders [4].

Most researchers suggested that foreign breeds, including Holsteins contributed little in the development of the Yaroslavl and Kholmogor cattle populations [6, 7], which was subsequently confirmed by molecular genetics studies [8–10]. The studies of the historical specimens of Yaroslavl and Kholmogor cattle, dated from the end of 19^th^ to the first half of 20^th^ century using microsatellites, indicated that Russian breeds and Holsteins were developed from different ancestral populations [11]. In the 1960s, the number of Kholmogor and Yaroslavl cattle was close to million individuals (993 and 951 thousand, respectively) each; in contrast, in 2015, their census population size remarkably decreased to 222.9 and 50.5 thousand heads, respectively [12]. During the last decade, along with the remarkable decrease in population size, crossbreeding has been actively used in the remaining purebred animals, which could lead to the loss of genetic distinctness.

Several molecular genetic studies have attempted to characterise the demographic history and population structure of the modern populations of Yaroslavl and Kholmogor breeds and their relationship to other taurine breeds [9, 10, 13, 14], but their results were, in some cases, controversial. By using microsatellite-based clustering, Li and Kantanen [13] suggested the composite origin of these breeds. Whole-genome studies performed using medium-density SNP chips showed that Yaroslavl and Kholmogor cattle are valuable national genetic resources, which have been little influenced by introgression with non-Russian breeds [9, 10]. The maintenance of a visible part of historical components in the modern populations both of Yaroslavl and Kholmogor breeds was confirmed by the study of historical specimens [11]. Further elucidation of the genetic architecture of these two breeds and more precise identification of genomic regions that are affected by putative selection may be achieved by using more powerful molecular genetic tools such as bovine high-density SNP BeadChip (Illumina Inc., USA) which contains 777,962 SNPs.

Several studies have explored the genome diversity and adaptation of cattle breeds by using high-density DNA arrays [15–17]. However, the patterns of genetic variation within Russian cattle breeds have not yet been assessed using this powerful genetic tool. Identification of the genome regions exhibiting evidence of being subjected to selective pressure during the breeding process is of particular interest [18]. Detailed analysis of these ‘signatures of selection’ can help to elucidate the breed history and to identify genes responsible for the adaptive and economically significant traits in livestock populations [19].

Studies have highlighted the importance of utilising multiple methodologies for detecting regions of the genome that have been the target of selection [20], including fixation index (*F*_ST_), hapFLK analysis and identification of runs of homozygosity (ROH) islands. *F*_ST_ quantifies the differences in allele frequencies between populations [21, 22] and is routinely used for identifying highly differentiated alleles [23, 24]. The hapFLK statistics is a haplotype-based method for the detection of positive selection in multiple population data [25]. ROH islands are genomic regions with high homozygosity around the selected locus that might harbour targets of positive selection [26–28].

Several studies have focused on identifying signatures of selection in transboundary and local cattle populations by using high-throughput SNP genotypes and *F*_ST_ [29–31], hapFLK, and ROH statistics [32]. However, only a limited number of studies have thus far attempted to identify signatures of selection in Russian cattle breeds, and they were based on the medium-density SNP genotypes [33, 34].

In this study, we performed the comprehensive analysis of high-density SNP genotypes to characterise the population structure and detect the signatures of selection in two old Russian cattle breeds (Yaroslavl and Kholmogor), by using Holsteins as the reference. The three methods (*F*_ST_, hapFLK, and ROH) were implemented to detect the genome regions that could be under putative selection. We identified the regions revealing signatures of selection in each of the two breeds, annotated the genes localised within or close to the selected regions, and performed a functional study of these genes. Our results might be useful for the genetic improvement of Yaroslavl and Kholmogor cattle breeds and developing programs for the conservation of their biodiversity.

## Materials and methods

### Ethics statement

This study does not involve any endangered or protected species. Samples were derived in part from the Bioresource Collection of the L.K. Ernst Federal Science Center for Animal Husbandry, supported by the Russian Ministry of Science and Higher Education. Blood samples (5 ml of whole blood) were collected for routine diagnostic purposes by trained personnel under strict veterinary rules. Sperm samples were provided by the artificial insemination (AI) stations according to specific scientific collaboration agreements.

The genotyping data supporting the results of this study are deposited in the Dryad Digital Repository (https://doi.org/10.5061/dryad.vt4b8gtq9).

### Sampling and DNA extraction

The pedigree records were analysed to identify the purebred animals of Yaroslavl (YRSL) and Kholmogor (KHLM) breeds (S1 Fig).

Only individuals having purebred ancestors on the both sides of pedigrees at least in three generations were selected for the analyses. To cover more breed variability, we sampled the animals originating from different breeding farms and representing different sire lines for each of the two breeds. Blood or semen samples were collected from 56 animals, including 31 samples of Yaroslavl breed and 25 samples of Kholmogor breed. DNA was extracted using Nexttec columns (Nexttec Biotechnology GmbH, Germany), according to manufacturer’s instructions. The concentrations of dsDNA solutions were measured using a Qubit 3.0 fluorometer (Thermo Fisher Scientific, Wilmington, DE, USA). The purity of DNA samples was checked by determining the OD_**260/280**_ ratio by using NanoDrop-2000 (Thermo Fisher Scientific, Wilmington, DE, USA). In addition, the DNA quality was checked using 1% agarose gel electrophoresis.

### SNP genotyping and quality control

The Illumina Bovine HD BeadChip (Illumina, San Diego, CA, USA) was used for genotyping. High-density SNP genotypes for European Holsteins (n = 25) obtained from Bahbahani et al. [16, 35] were included in the dataset as the reference. R software [36] was used to create input files. Valid genotypes for each SNP were determined by setting the cut-off of 0.5 for the GenCall (GC) and GenTrain (GT) scores [37]. PLINK 1.9 software [38] was used to perform SNP quality control. All animals were subjected to filtering for genotyping efficiency (—mind 0.1). The SNPs genotyped in less than 90% of the samples (—geno 0.1) and those located on sex chromosomes were excluded from the analysis. The final data set used for analysis included 650,134 autosomal SNPs. Additional filters for linkage disequilibrium (LD) and minor allele frequency (MAF) were used to calculate several statistical parameters (see below). The positions of SNPs were assigned according *Bos taurus* genome assembly UMD 3.1.1 (https://www.ncbi.nlm.nih.gov/assembly/GCF_000003055.6).

### Genetic diversity

Within-population genetic diversity was evaluated using 133,685 SNPs after LD-based pruning by using PLINK 1.9 [38]. One locus from each SNP pair where the LD (r^**2**^) exceeded 0.5 within 50 SNP windows was removed (—indep-pairwise 50 5 0.5 flag, where 50 is the size of the sliding window, 5 is the number of SNPs shifted in each step, and 0.5 is the r^**2**^ threshold). We calculated the observed heterozygosity (*H*_**O**_), unbiased expected heterozygosity (_**U**_*H*_**E**_) [39], rarefied allelic richness (*A*_**R**_), and inbreeding coefficient (_**U**_*F*_**IS**_) based on the unbiased expected heterozygosity by using R package “diveRsity” [40]. The genomic inbreeding coefficient based on ROH (*F*_**ROH**_) was computed as the ratio of the sum of the length of all ROHs per animal to the total autosomal SNP coverage (2.51 Gb; for ROH estimation, see section ‘Runs of homozygosity’ below).

### Effective population size

Trends of effective population size were estimated from LD by using the SNeP tool, as described by Barbato et al. [41]. We applied default parameters, except for sample size correction, mutation occurrence (α = 2.2) [42], and recombination rate between a pair of genetic markers, according to Sved and Feldman [43]. To determine the rate of *N*_**E**_ changes in the 50 most recent generations, we determined the *N*_**E**_ changing ratio (*N*_**E**_C) by calculating the slope of each segment that links a pair of neighbouring *N*_**E**_ estimates and normalising the values by using the median of the most recent 50 *N*_**E**_ estimates. Only SNPs with an MAF of >0.05 were used to estimate the effective population size.

### Runs of homozygosity

A window-free method for consecutive SNP-based detection (consecutive runs method [44] implemented in R package “detectRUNS” [45] was used for the estimation of ROHs. We allowed one SNP with missing genotype and up to one possible heterozygous genotype in one run to avoid the underestimation of the number of ROHs that were longer than 8 Mb [46]. The minimum ROH length was 1000 kb. For minimising false-positive results, we calculated the minimum number of SNPs (l) as was initially proposed by Lencz et al. [47] and then by Purfield et al. [48]:

l=loge(α/ns*ni))/(loge(1‐het),where

ns = the number of genotyped SNPs per individual; ni = the number of genotyped individuals; *α* = the percentage of false-positive ROHs (set to 0.05 in our study), and het = the mean heterozygosity across all SNPs. In our case, the minimum number of SNPs was 31.

The ROH number was estimated for each individual and then distributed between the following ROH length classes: (1, 2), (2, 4), (4, 8), (8, 16), and >16. To estimate the proportion of genome covered by different ROH segments, we calculated the sum of ROHs for different minimum lengths (>1, >2, >4, >8, or >16 Mb). The number and length of ROHs were averaged per animal within each breed.

### Principal component analysis, Neighbor-Net, and admixture

To characterize the relationship within and between populations at the genomic level and to assess the individuals with regard to their assignment to the corresponding breed, we performed principal component analysis (PCA), Neighbor-Net clustering, and model-based clustering (admixture). PLINK v1.9 software was used to perform PCA, and R package “ggplot2” was used to visualise the results [49]. A Neighbor-Net tree was constructed on the basis of pairwise identical-by-state (IBS) distances in SplitsTree4 [50]. We employed Admixture v1.3 [51] for genetic admixture and R package “pophelper” [52] for plotting the results. The number of assumed ancestral populations (K) was estimated on the basis of the lowest cross-validation (CV) error compared to other K values by using a standard admixture cross-validation procedure [53].

### Selection signature analysis

We used three different statistics for detecting the signatures of selection in the genome of the studied cattle populations: calculation of the fixation index (*F*_**ST**_) for each SNP for comparison of the pairs of breeds, hapFLK analysis, and estimation of the ROH islands, which were overlapped among different animals within each breed.

We calculated pairwise genetic differentiation (*F*_**ST**_) [53] between all SNPs in pairs of the studied cattle breeds (KHLM/HLST, YRSL/HLST, and KHLM/YRSL) by using PLINK 1.9. We applied a low threshold for MAF of less than 5% (—maf 0.05), because the filtering of SNPs based on MAF may affect the probability of identifying alleles related to selection [33]. The data set used for *F*_**ST**_ analysis included 567,206 autosomal SNPs. The top 0.1% *F*_**ST**_ values were used to represent the selection signature, as was previously considered by Kijas et al. [18] and Zhao [24].

The hapFLK method considers the haplotype structure of the population [25]. We used hapFLK 1.4 program [25] to detect the signatures of selection through haplotype differentiation among the studied populations. The number of haplotype clusters per chromosome was determined in fastPHASE by using cross-validation-based estimation and was set at 25 [54]. For detailed analyses, we selected the hapFLK regions containing at least one SNP with *p*-value threshold of 0.001 (-log10(*p*) > 3). The hapFLK regions carrying at least one SNP with *p*-value threshold of 0.00001 (-log10(*p*) > 5) were defined as the strongest hapFLK regions. To limit the number of false positives, we calculated q-values by using R package qvalue [55]. We applied a q-value threshold of 0.05 (corresponds to a false discovery rate of 5%).

We used R package “detectRUNS” [45] for ROH estimation. We selected the overlapping ROH segments with the minimal ROH length of 0.3 Mb shared by more than 50% of the samples as an indicator of possible ROH islands in the genome. Special attention was paid to the ROH islands shared by more than 70% of the samples, which were defined as the strongest ROH islands.

The results obtained by the three different methods (*F*_**ST**_, hapFLK, and ROH) were compared, and genomic regions selected by at least two different methods were identified. Moreover, we compared our results with the data previously obtained by Yurchenko et al. [34] by using de-correlated composite of multiple signals (DCMS) statistics [56] for the presence of the overlapping genomic regions. Besides, we compared the identified genomic regions with the meta-assembly of selection signature in cattle [57].

### Identification of genes and gene function within selected regions of the genome

The *F*_**ST**_ statistic windows of 0.4 Mb (0.2 Mb upstream and 0.2 Mb downstream of the top 0.1% SNPs) were investigated to select overlapping gene segments. For hapFLK and ROH analyses, the genes were selected if they were localised entirely or in part within the identified genomic regions. Gene identification was performed using *Bos*_*taurus*_UMD_3.1.1 genome assembly (**https://www.ncbi.nlm.nih.gov/assembly/GCF_000003055.6**). The selected genes were considered as candidate genes. In addition, genes were compared with quantitative trait locus (QTL) regions included in the Cattle QTL database (**http://www.animalgenome.org/cgi-bin/QTLdb/index**) [58]. Phenotypes that are known to be associated with the identified candidate genes were inferred from the most recent literature, by integrating PubMed database of the National Center of Biotechnological Information (**https://pubmed.ncbi.nlm.nih.gov/**).

The information for the annotation of some specific genes was sourced from Gene Cards (**http://www.genecards.org/**).

## Results

### Genetic diversity

The estimation of the average heterozygosity within each breed indicated significant genetic diversities among the studied breeds (Table 1).

**Table 1:** Summary statistics for the studied breeds calculated on the basis of the high-density SNP genotypes.

Breed	Acronym	n	*H*_O_	_U_*H*_E_	_U_*F*_IS_ [CI 95%]	*F*_ROH_	*A*_R_	*N*_E5_
Yaroslavl	YRSL	31	0.354±0.0001	0.349±0.0001	-0.013 [-0.014; -0.012]	0.103±0.006	1.967±0.0001	111
Kholmogor	KHLM	25	0.370±0.0001	0.361±0.0001	-0.022 [-0.023; -0.021]	0.059±0.003	1.977±0.0001	130
Holstein	HOL	25	0.358±0.0001	0.354±0.0001	-0.01 [-0.011; -0.009]	0.108±0.006	1.966±0.0001	95

Note: n, the number of individuals; *H*_O_, observed heterozygosity; _U_*H*_E_, unbiased expected heterozygosity; _U_*F*_IS_, the inbreeding coefficient; *F*_ROH_, inbreeding coefficient, calculated on the basis of ROH; *A*_R_, allelic richness; *N*_E5_, effective population size 5 generations ago.

The average level of _U_*H*_E_ for the Yaroslavl breed was lower, whereas that for the Kholmogor breed was higher than that of Holsteins. The calculation of _U_*F*_IS_ indicated heterozygote excess in all populations beyond the expected number of heterozygotes. The _U_*F*_IS_ values were similar for Yaroslavl and Holstein breeds, whereas the Kholmogor breed had higher heterozygote excess. The estimation of inbreeding coefficients based on *F*_ROH_ showed a similar pattern. That is, the *F*_ROH_ values for Yaroslavl and Holstein breeds were significantly higher than those of the Kholmogor breed (0.103 and 0.108 vs 0.059, *p* < 0.001). Higher *F*_ROH_ values are associated with increased degree of inbreeding. We did not observe differences in allele richness (*A*_R_) between Yaroslavl and Holstein breeds; in contrast the Kholmogor breed had higher *A*_R_ values.

### Effective population size

**Fig 1:**
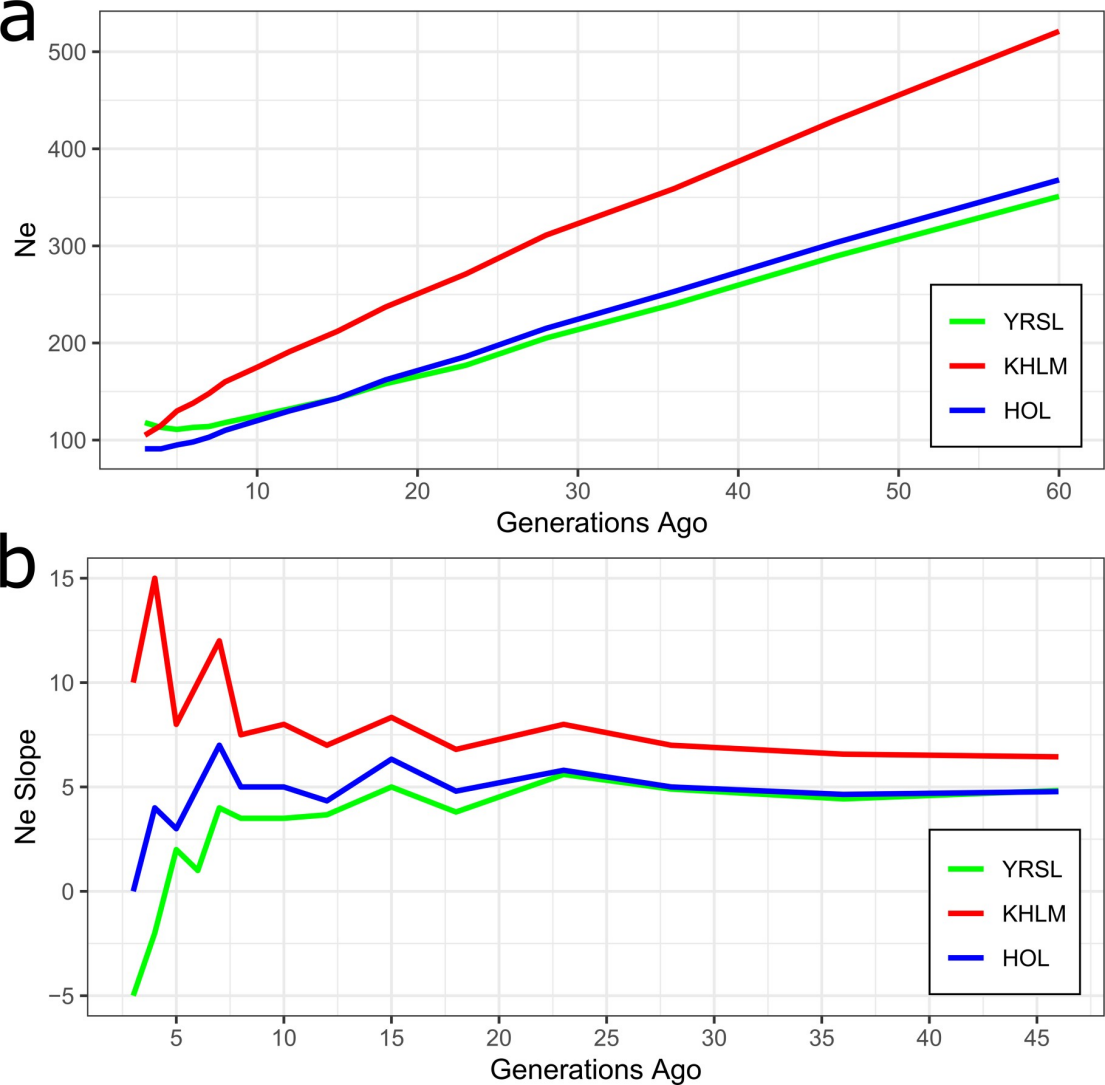
The effective population size (*N_E_*) across generations from approximately 50 generations ago based on linkage disequilibrium (LD) calculations (a) and N_E_ slope (b). Note: Breed: YRSL, Yaroslavl; KHLM, Kholmogor; HOL, Holsteins.

The Yaroslavl and Kholmogor breeds had higher effective population size (*N*_E5_ = 111 and 130, respectively) than that of Holsteins (*N*_E5_ = 95) (Table 1). The *N*_E_ values for Kholmogor breed declined over time, whereas the lowest *N*_E_ value was observed for Yaroslavl breed 5 generations ago (*N*_E_ = 111) with the subsequent increase to 118 at 3 generation ago (Fig 1A, S2 Fig).

All the three studied breeds had a similar pattern of the *N*_E_ slope changes up to 7 generations ago. We observed 3 common peaks in *N*_E_ changes characterised by accelerated decline of the effective population size 23, 15, and 7 generations ago. The most recent peak in *N*_E_ for Kholmogor breed was found 4 generations ago (Fig 1B).

### Estimates of ROHs and *F*_ROH_

**Fig 2:**
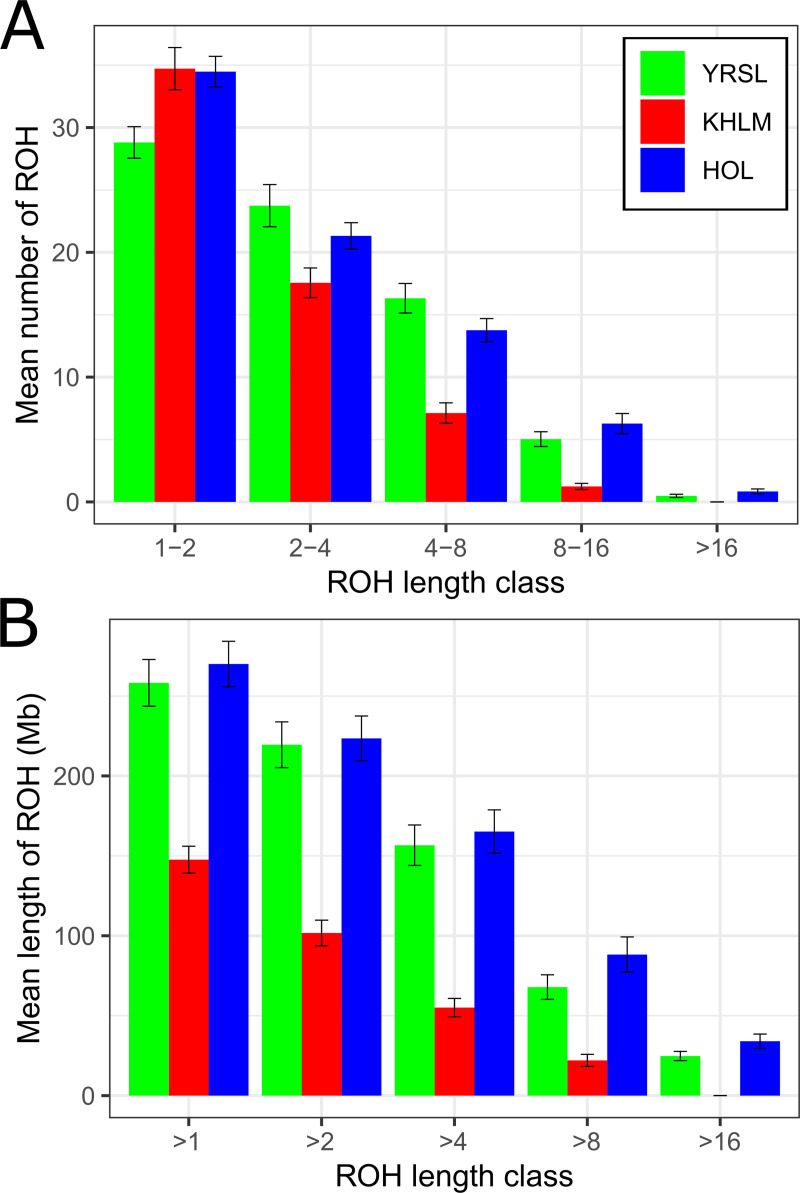
Descriptive statistics of runs of homozygosity (ROH) by ROH length class. Note: Breed: YRSL, Yaroslavl; KHLM, Kholmogor; HOL, Holsteins. (A). Number of ROHs by ROH length class: axis X, ROH classes (1–2 Mb, 2–4 Mb, 4–8 Mb, 8–16 Mb, and >16 Mb); axis Y, mean number of ROHs; (B). Length of ROHs by ROH length class: axis X, ROH classes (>1 Mb, >2 Mb, >4 Mb, >8 Mb, and >16 Mb); axis Y, mean length of ROHs.

The ROH segments were found in all the studied breeds, with an average number of 71.84 ± 3.27 segments in Yaroslavl breed and 58.96 ± 2.38 segments in Kholmogor breed and mean ROH length of 3.59 and 2.50 Mb, respectively. In Holsteins, the values of the above-mentioned estimations were 74.60 ± 2.28 segments and 3.62 Mb, respectively. Short segments (ROH_1–2 Mb_), caused by common ancestors around 25 to 50 generations ago, were the most distributed through the genome and accounted for 38.73% in Yaroslavl breed and 57.26% in Kholmogor breed of all ROHs identified, although the proportion of the genome covered by ROHs of this length class was relatively small (15.0% and 31.1%, respectively). In Holsteins, 44.97% of ROHs were characterised by the shortest ROH segments, which covered 17.2% of the genome (Fig 2, S1 Table).

The proportion of ROH segments with the greatest length (>16 Mb), typically caused by inbreeding to a very recent ancestor, as in parent–offspring, half-sib mating, or first cousin mating, was extremely low in the Yaroslavl and Holstein breeds (0.65% and 0.10% of the total number of ROHs, respectively). The genome coverage by the longest ROHs was the lowest and averaged 9.6% in the Yaroslavl breed. We did not find long ROH segments (>16 Mb) in the Kholmogor breed.

### Breed relationship and admixture

The results of PCA revealed well-separated clusters corresponding to each of the three breeds. The first component, which explained 7.02% of the genetic variability, split the Yaroslavl breed from the Kholmogor breed and Holsteins, suggesting an early divergence of Yaroslavl from old Friesians; in contrast, the Kholmogor breed was separated from Holsteins by the second component, which was responsible for 5.03% of diversity (S3A Fig).

A Neighbor-Net tree based on pairwise IBS distances showed breed-specific distribution of individuals between three branches, which joined individuals of the same breed (S3B Fig). To identify ancestry and admixture level among the studied breeds, we performed admixture analysis for the number of clusters (*K*) from 1 to 5 (S3C Fig). At *K* = 2, the Yaroslavl breed was clearly divided from Holsteins, confirming its earlier divergence. At *K* = 3 (corresponding to the minimal value of CV error; S3D Fig), each of the three breeds formed their own clusters. Only slight level of admixture was observed in the several animals of Yaroslavl breed, whereas the Kholmogor breed was characterised by slightly greater level of admixture.

### *F*_ST_ statistics

**Fig 3:**
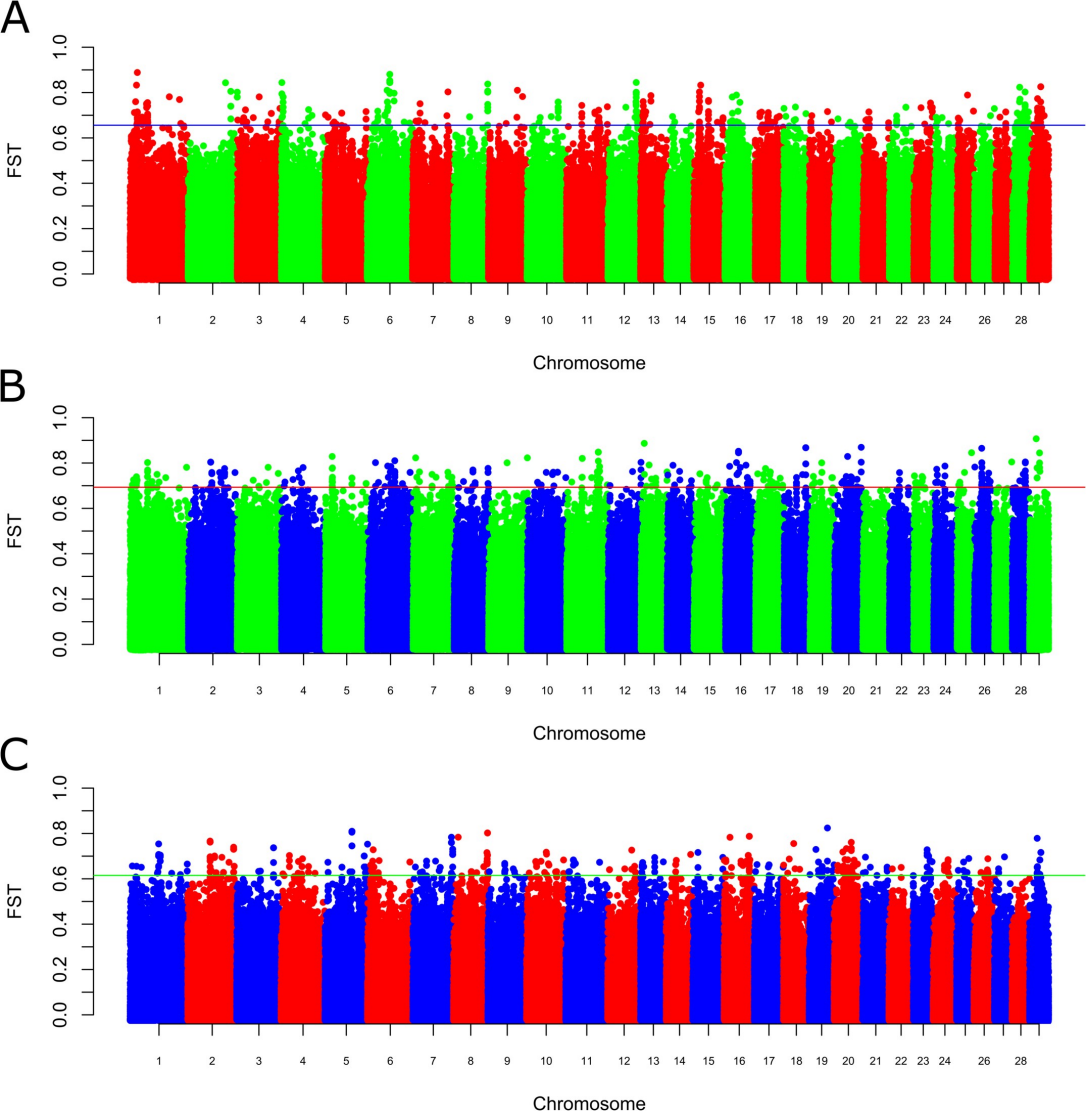
Genomic distribution of *F*_ST_ values estimated between pairs of breeds. (A). Yaroslavl and Kholmogor breeds. (B). Yaroslavl and Holstein breeds. (C). Kholmogor and Holstein breeds. Note: Axis X, cattle autosomes (the breadth of autosomes corresponds to their length); axis Y, *F*_ST_ values. SNPs were plotted relative to their positions within each autosome. The horizontal line on each plot indicates the threshold, which was estimated as the top 0.1% for *F*_ST_ values. The SNPs with *F*_ST_ beyond the cut-off value were considered to be under selection pressure.

We observed variation in genetic differentiation between breeds, based on *F*_ST_ through the genome (Fig 3A–3C).

**Fig 4:**
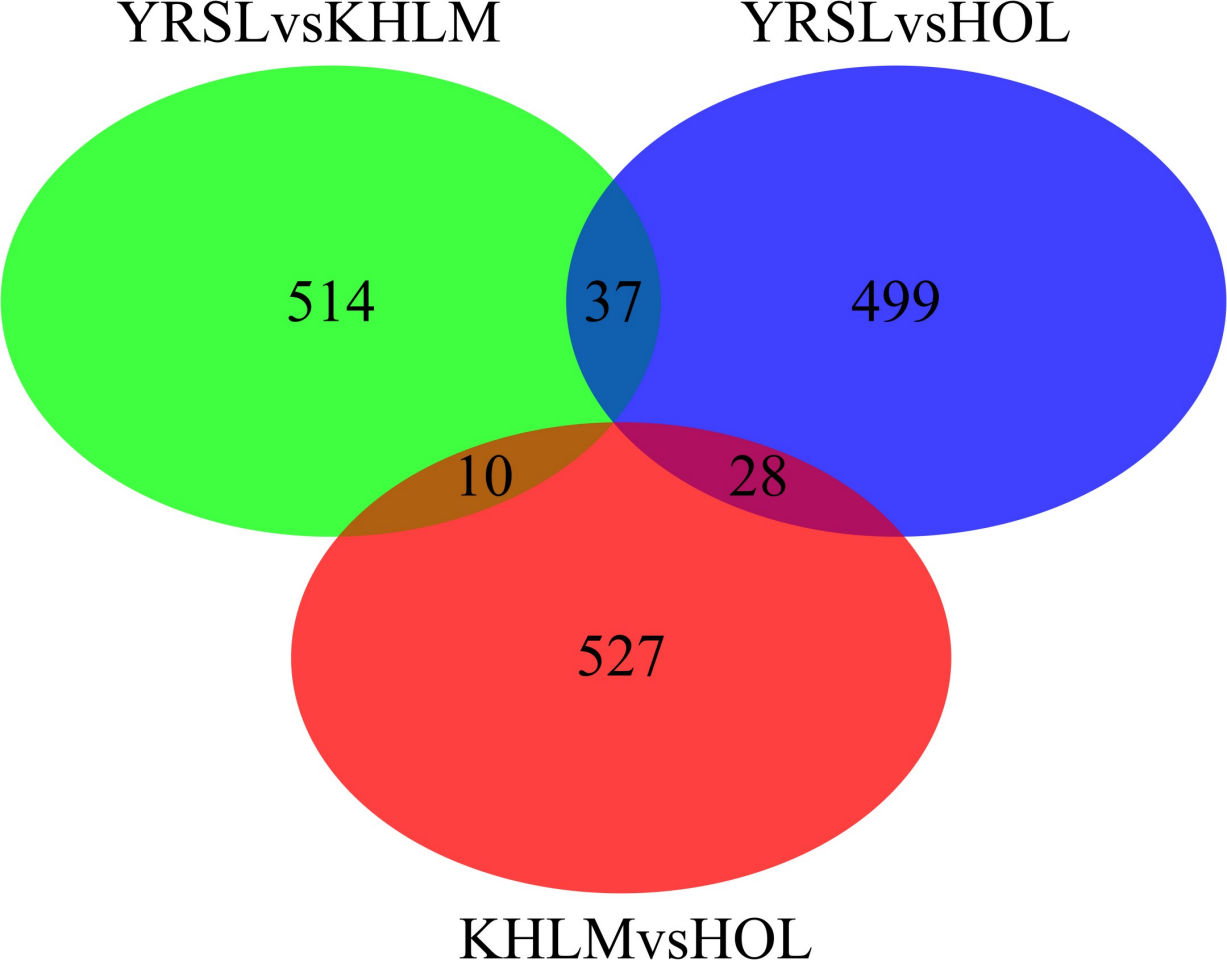
Venn diagram illustrating the distribution of the top 0.1% of SNPs by *F*_ST_ value between pairs of breeds. Note: Breed: YRSL, Yaroslavl; KHLM, Kholmogor; HOL, Holsteins.

The average pairwise *F*_ST_ values between breeds were 0.090 for Yaroslavl and Kholmogor breeds, 0.107 for Yaroslavl and Holstein breeds, and 0.081 for Kholmogor and Holstein breeds. We identified 561, 564, and 565 SNPs with *F*_ST_ beyond the cut-off value for the above-mentioned pairs of breeds, respectively, and thus considered them to be under selection pressure (S2 Table). The selected SNPs included single SNPs as well as SNP blocks consisting of several neighbour SNPs. The greatest number of top 0.1% SNPs for YRSL/HOL comparison was obtained on chromosomes 2, 16, 6, 11, 1, 26, 5, 20, 17, and 18 (74, 45, 39, 37, 33, 33, 30, 30, 28, and 24 SNPs, respectively), and the lowest was obtained on chromosomes 25, 22, 9, 27, and 21 (5, 3, 2, 1, and 0 SNPs, respectively). The KHLM/HOL comparison revealed the greatest number of top 0.1% SNPs on chromosomes 20, 8, 29, 2, 7, 5, 16, 1, 10, and 6 (60, 59, 48. 46, 34, 30, 30, 27, 26, and 25 SNPs), whereas the lowest were on chromosomes 25, 22, and 28 (5, 3, and 0 SNPs, respectively). Although the distribution of selected top 0.1% SNPs was breed specific in most cases, we identified a small number of SNPs which were under selection pressure in two pairs of breeds, including 37 SNPs for YRSL/KHLM and YRSL/HOL, 28 SNPs for YRSL/HOL and KHLM/HOL, and 10 SNPs for KHLM/HOL and YRSL/KHLM (Fig 4, S3 Table).

### The hapFLK analysis

**Fig 5:**
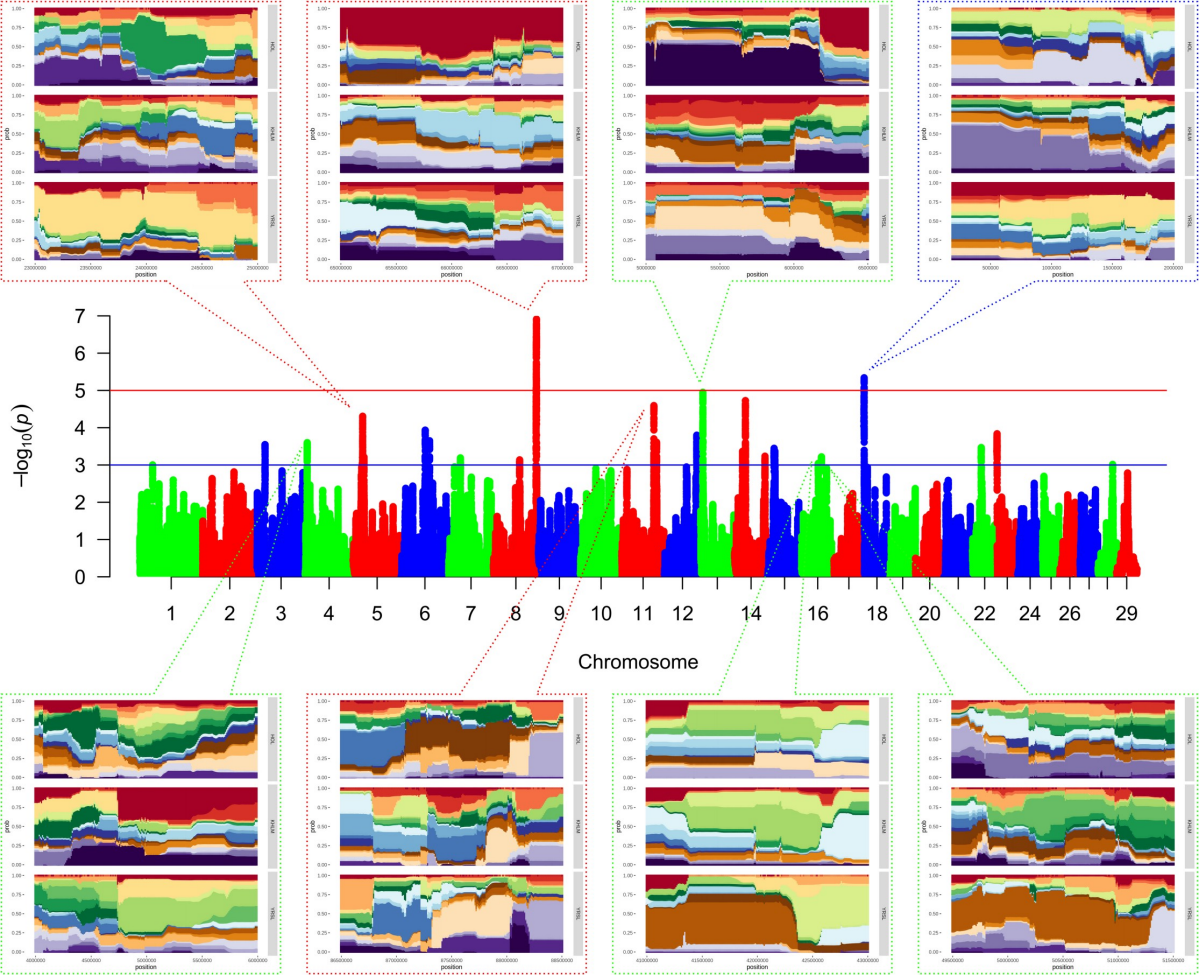
Genome-wide scan for the signature of selection based on the hapFLK statistics. Note: Each dot corresponds to a single SNP; axis X, genomic coordinates by chromosome; axis Y, statistical significance (−log10 *P*-values); the red and blue lines indicate the thresholds of significance: 10^−5^ and 10^−3^, respectively; the figures above and below the plot present the magnified plots of the chromosome areas containing the hapFLK regions.

The hapFLK analysis revealed 24 putative regions affected by selection, which were distributed among 16 autosomes (S4 Table, Fig 5).

Thirteen hapFLK regions were identified in the Yaroslavl breed, 6 in the Kholmogor breed, and 12 in Holsteins. In most cases, the chromosomal distribution of these regions was breed-specific. The regions identified by the hapFLK analysis were observed on 9 autosomes (BTA4, BTA5, BTA6, BTA11, BTA13, BTA14, BTA15, BTA16, and BTA22) in Yaroslavl breed and on 6 autosomes (BTA4, BTA6, BTA12, BTA15, BTA18, and BTA23) in Kholmogor breed. For comparison, the hapFLK regions were detected on nine autosomes (BTA1, BTA3, BTA7, BTA8, BTA11, BTA13, BTA14, BTA22, and BTA23) in Holsteins. Both Yaroslavl and Kholmogor breeds had hapFLK regions overlapping with Holsteins (4 and 1 region, respectively) and with each other (2 regions). The sizes of the putative regions to be under selection pressure ranged from 0.336 to 2.974 Mb in Yaroslavl breed, from 0.284 to 1.266 in Kholmogor breed, and from 0.080 to 2.974 in Holsteins.

### Identification of ROH islands

Overlapping ROH islands observed in more than 50% of samples within each breed were found across the genome in all the studied breeds (S5 Table, S4 Fig).

We identified 17 and 20 ROH islands which were distributed among 12 chromosomes in the genome of Kholmogor and Yaroslavl cattle breeds, respectively. In most cases, the genomic distribution of ROH islands was specific for each breed both in length and localisation across chromosomes. The lengths of ROHs varied from 304.8 to 1276.9 kb in Kholmogor breed and from 297.2 to 1640.6 kb in Yaroslavl breed and were averaged 638.1 ± 59.4 and 691.0 ± 71.6 kb, respectively.

For comparison, in Holsteins, 29 ROH islands were identified, which were distributed among 16 autosomes. The mean ROH length was 735.6 ± 85.8 kb with variation from 304.8 to 2741.3 kb. We identified several overlapping ROHs, which were common for two or three breeds. The ROHs located on BTA3 (positions from 9,205,782 to 9,515,045), BTA6 (positions 5,252,711 to 6,502,610), and BTA14 (positions 24,419,295 to 25,066,322) were common for all the three studied breeds. The ROH regions on BTA7 (positions 52,077,420 to 52,572,307), BTA13 (positions 50,231,261 to 50,724,998), BTA16 (positions 42,691,909 to 43,535,617), and BTA29 (positions 38,947,760 to 39,252,602) were common for Kholmogor and Holstein breeds. The common ROH islands on BTA12 (positions 72,509,035 to 73,492,233), BTA16 (positions 43,804,119 to 44,269,629), and BTA21 (positions 2,082,072 to 2,482,464) were observed in the genome of Yaroslavl and Holstein breeds. Among the described ROH islands, we identified 6 strongest ROHs (present in more than 70% of samples) in the genome of Yaroslavl breed on BTA5 (positions 24,567,855 to 24,934,918), BTA6 (positions 5,252,711 to 6,522,730), BTA7 (positions 20,531,857 to 21,146,198), BTA16 (positions 39,698,386 to 40,620,308 and 42,507,253 to 43,049,359), BTA17 (35,494,057 to 36,118,075), and BTA21 (positions 2,082,072 to 2,504,481) as well as 3 strongest ROHs in the genome of Kholmogor breed on BTA7 (positions 51,178,057 to 51,555,664), BTA14 (positions 24,562,756 to 24,874,608), and BTA16 (positions 42,691,909 to 43,472,069). One strongest ROH region common for Kholmogor and Holstein breeds and one region common for Yaroslavl and Holstein breeds was found on BTA14 and BTA16 (S5 Table).

Comparison of the genomic localisation of the regions identified in our study by using *F*_ST_, hapFLK, and ROH analyses, as well as in the previous study performed by Yurchenko et al. [34] by using DCMS statistic [56] showed the presence of several genomic regions under putative selection in the studied breeds identified at least by two different methods (Table 2).

**Table 2:** Genomic regions under putative selection in the two old Russian cattle breeds compared to those in Holsteins identified at least by two different methods.

BTA	Breed	Genomic regions (Mbp) under selection identified by different methods			
		*F*_*ST*_ ^1^	*hapFLK*^1^	*ROH50*^1^	*DCMS*^2^
1	HOL	32.45–32.51	32.45–32.53		
	YRSL			65.31–65.61	65.25–65.69
	HOL	84.20–84.23		83.68–84.55	83.86–84.48
2	HOL	101.86–102.34		101.68–102.16	101.84–102.00
3	HOL			8.97–9.72	9.39–9.53
	HOL			53.89–54.25 54.26–54.33	53.81–54.80
4	YRSL	5.01–5.11	4.74–5.36		
5	YRSL		23.76–24.40	24.48–25.26	23.95–26.34
6	KHLM	60.34–60.70	60.05–60.74		60.30–60.76
	YRSL	71.46–71.55	71.38–72.04		71.24–71.96
7	YRSL			10.22–10.69	10.07–10.29
	YRSL			20.53–21.19	20.63–20.98
	HOL			43.01–43.61	43.43–43.57
	HOL			46.96–47.97	47.38–47.74
	HOL			52.08–52.60	51.57–52.42
8	HOL			59.67–60.32	59.81–60.21
	HOL	107.80 – 108.58	107.44–109.46^a^	107.62–108.84	107.76–108.68
9	KHLM			64.53–65.13	64.86–65.11
12	KHLM	81.45–81.58	81.40–81.69		
13	HOL, YRSL	5.18–5.67	4.97–6.82		5.28–6.40
14	KHLM			1.54–2.09	1.70–1.89
	HOL	21.12		21.10–21.58	
	HOL, YRSL, KHLM			24.42–25.07	24.43–25.10
	HOL		28.58–28.69	28.34–29.17	
15	KHLM	16.13, 16.14	16.04–16.62		
	YRSL	18.03, 18.25	17.80–18.77		
16	YRSL		41.91–42.48	42.48–43.13	42.43–43.37
	HOL			42.56–43.54	42.79–43.50
	KHLM			42.69–43.54	42.87–43.17
17	YRSL	45.60		45.59–45.61	
18	KHLM	0.85, 0.88	0.19–1.46		
	KHLM			14.04–14.98	14.12–14.99
24	YRSL	41.74		41.76–41.89	41.67–42.18
26	HOL			19.53–20.28	19.19–20.29
	HOL	21.20–22.91		22.13–23.01	21.53–22.95
29	HOL			38.67–38.92 38.95–39.25	38.41–39.33

Note: BTA, *Bos taurus* autosome; Breed: YRSL, Yaroslavl; KHLM, Kholmogor; HOL, Holsteins; genomic regions: start and end positions (Mbp) according to *Bos*_*taurus*_UMD_3.1.1 genome assembly (https://www.ncbi.nlm.nih.gov/assembly/GCF_000003055.6); methods used for the identification of the signature of selection: *F*_*ST*_, top 0.1% SNPs by *F*_ST_ value at pair-wise breed comparison; *hapFLK*, regions identified by hapFLK analysis; *ROH50*, ROH segments distributed in more than 50% of animals within each of the studied breed

^1^present study

^2^Yurchenko et al. [34]; pairs of breeds, used for *F*_ST_ calculations

^a^KHLM/HOL

^b^YRSL/HOL

^c^YRSL/KHLM

^d^weak selection signal was also observed in Kholmogor breed.

Twelve such regions were detected in Yaroslavl breed and nine in Kholmogor breed compared to 17 regions in Holsteins.

### Identification of candidate genes within selected regions of the genome

We identified candidate genes within which the SNPs selected by *F*_ST_ analysis were localised (S2 Table), as well as the genes entirely or in part localised within the detected hapFLK regions and ROH islands (S6 Table). For more thorough analysis, we selected the genes, localised within genomic regions, which revealed the signature of selection identified by hapFLK and/or carried the strongest ROHs (ROH islands observed in more than 70% of animals) or were detected at least by two different methods (Table 3).

**Table 3:** Genes localised within the genomic regions affected by putative selection in Yaroslavl and Kholmogor cattle breeds.

BTA	Region (Mb)	Breed	Methods	Genes
1	65.3 – 65.7	YRSL	ROH50, DCMS^1^	*GSK3B*, *GPR156*
4	4.7 – 5.4	YRSL	*F*_ST_, hapFLK	*Grb10*, DDC*
5	24.5 – 25.3	YRSL	hapFLK, ROH, DCMS	*PLXNC1*, *CCDC41*, *TMCC3*, ***NDUFA12*****,** ***FGD6***, *VEZT*, *MIR331*
6	5.2 – 6.5	YRSL	ROH	***MAD2* ,** ***MAD2L1*****, *MGC134093***
6	60.1 – 60.8	KHLM	*F*_ST_, hapFLK, DCMS	*WDR19*, KLB*, RPL9*, LIAS*, UGDH*, *UBE2K*, *PDS5A*
6	71.4 – 72.1	YRSL	*F*_ST_, hapFLK, DCMS	PDGFRA*, *KIT*
7	10.2 – 10.7	YRSL	ROH, DCMS	
7	20.5 – 21.2	YRSL	ROH, DCMS	***TICAM1*****, *FEM1A*, *MIR7*, *DPP9*, *TNFAIP8L1*, *SEMA6B*,** ***PLIN5*****, *LRG1*,** ***PLIN4*****, *HDGF2UBX*, *N6*, *CHAF1A*, *SH3GL1*, *STAP2*, *MPND*, *FSD1*, *TMIGD2*, *SHD*, *CCDC94*, *EBI3*, *ANKRD24*,** ***SIRT6*****,** ***CREB3L3*****, *MARP2K2***
7	51.2 – 51.6	KHLM	ROH	***NME5*****, *BRD8*, *MIR2459*, *GFRA3*, *KIF20A*, *CDC23*, *FAM53C*, *CDC25C*, *REEP2*,** ***EGR1*****, *ETF1*,** ***HSPA9***
9	64.5 – 65.1	KHLM	ROH, DCMS	*SYNCRIP*, *NT5E*
12	81.4 – 81.7	KHLM	*F*_ST_, hapFLK	*NALCN*
13	5.0 – 6.8	YRSL^a^	*F*_ST_, hapFLK, DCMS	BTBD3*
14	1.5 – 2.1	KHLM	ROH, DCMS	*LRRC24*, *LRRC14*, *RECQL4*, *MFSD3*, *GPT*, *PPP1R16A*, *FOXH1*, *CYHR1*, *NFKBIL2*, *SLC39A4*, *CPSF1*, *ADCK5*, *GPR172B*, *FBXL6*, *SCRT1*, *DGAT1*, *HSF1*, *BOP1*, *SCXB*, *HEATR7A*, *MAF1*, *SHARPIN*, *CYC1*, *GPAA1*, *EXOSC4*, *OPLAH*, *GRINA*, *PARP10*, *MIR23089*
14	24.4 – 25.1	YRSL^a^	ROH, DCMS	***XKR4*****,** ***TMEM68*****,** ***TGS1***, *LYN*, *RPS20*, *MOS*, ***PLAG1*****,** ***CHCHD7***
14	24.4 – 25.1	KHLM^a^	ROH, DCMS	
15	16.0 – 16.6	KHLM	*F*_ST_, hapFLK	*PIWIL4*, FUT4*, MGC137061*, *CWF19L2*, *GUCY1A2*
	17.8 – 18.8	YRSL	*F*_ST_, hapFLK	*RAB39*, CUL5*, ACAT1*, *NPAT*, *ATM*, KDELC2*, *EXPH5*, *DDX10*
16	39.7 – 40.6	YRSL	ROH	***FMO1*, *FMO4*, *BAT2L2*, *MYOC*, *VAMP4*, *METTL13*, *MIR214*, *MIR199A1***
	42.5 – 43.1	YRSL^a^	hapFLK, ROH, DCMS	*VPS13D*, *TNFRSF1B*, *TNFRSF8*, ***MIIP*,** ***MFN2*****,** ***PLOD1*****,** ***KIAA2013*****,** ***NPPB*****,** ***NPPA*****,** ***CLCN6*****,** ***MTHFR*****,** ***AGTRAP*****, *MAD2L2*, *FBXO6*, *FBXO44*,** ***FBXO2*****, *PTCHD2***
	42.7 – 43.5	KHLM^a^	ROH, DCMS	***NPPB*****,** ***NPPA*****,** ***CLCN6*****,** ***MTHFR*****,** ***AGTRAP*****, *MAD2L2*, *FBXO6*, *FBXO44*,** ***FBXO2*****, *PTCHD2*, *UBIAD1*,** ***MTOR*****,** ***ANGPTL7*****,** ***EXOSC10*****, *SRM*,** ***MASP2***, *TARDBP*
17	35.5 – 36.1	YRSL	ROH	***IL2*****,** ***ADAD1***
18	0.2 – 1.5	KHLM	*F*_ST_, hapFLK	UQCRFS1*, *VSTM2B*, *VAC14*
	14.0 – 15.0	KHLM	ROH, DCMS	*CDT1*, *APRT*, *GALNS*, *TRAPPC2L*, *CBFA2T3*, *ACSF3*, *CDH15*, *MGC157263*, *ANKRD11*, *SPG7*, *RPL13*, *CPNE7*, *DPEP1*, *CHMP1A*, *CDK10*, *SPATA2L*, *ZNF276*, *FANCA*, *TCF25*, *SPIRE2*, *MC1R*, *TUBB3*, *MIR220D*, *DEF8*, *CENPBD1*, *DBNDD1*, *GAS8*, *SHCBP1*
21	2.0 – 2.5	YRSL	ROH	*UBE3A*
24	41.7 – 41.9	YRSL	*F*_ST_, ROH, DCMS	NDUFV2*

Note: BTA, *Bos taurus* autosome; region, start and end positions of the genomic region affected by putative selection (Mbp) according to *Bos*_*taurus*_UMD_3.1.1 genome assembly (https://www.ncbi.nlm.nih.gov/assembly/GCF_000003055.6); breed: YRSL, Yaroslavl; KHLM, Kholmogor; methods: *F*_*ST*_, top 0.1% SNPs by *F*_ST_ value at pair-wise breed comparison; *hapFLK*, regions identified by hapFLK analysis; *ROH*, ROH segments distributed in more than 50% of animals within each of the studied breed (genes localised within ROH segments identified in more than 70% of animals are shown by bold)

^1^the signature of selection identified previously by Yurchenko et al. [34] based on de-correlated composite of multiple signals (DCMS)

^a^genomic regions, which are common with Holsteins

*causative SNPs, detected by *F*_ST_ analyses were localized within 0,4 Mb windows; functionally annotated genes are underlined.

Functional annotation of the identified candidate genes showed the presence of genes associated with growth (localised on BTA 4, BTA 6, BTA 7, BTA 14, BTA 15, BTA 16, BTA 17, BTA 18, and BTA 21), carcass-related traits (BTA 6, BTA 7, BTA 15, and BTA 18), feed efficiency (BTA 14, BTA 15, and BTA 18), milk production (BTA 5, BTA 6, BTA 14, and BTA 16), and reproduction (BTA 1, BTA 4, BTA 6, BTA 7, BTA 9, BTA 15, BTA 16, BTA 17, and BTA 18), involved in the metabolic process (BTA 6, BTA 7, BTA 15, BTA 16, and BTA 24), and responsible for immunity (BTA 5, BTA 6, BTA 7, BTA 15, BTA 16, BTA 17, and BTA 18) and adaptation (BTA 18; S7 Table).

## Discussion

### Genetic diversity, effective population size, and breed relationship

To our knowledge, this is the first comprehensive genome-wide study of putative signatures of selection in the genomes of Yaroslavl and Kholmogor breeds performed on the basis of the analysis of high-density SNP genotypes (650,134 autosomal SNPs) generated using Bovine SNP HD BeadChip (Illumina, USA). In our recent studies, we showed that these two oldest Russian native breeds have been little influenced by introgression with transboundary breeds and maintained their historical genomic components [9, 11]; thus, they may carry specific signatures of selection, reflecting their adaptation to the local environmental conditions and response to the breeding strategy used. Adaptation to the changing environment is increasingly important [59]; thus, native cattle breeds might become valuable sources of genetic variability, which will be necessary for developing sustainable animal production systems in the future.

Considering the relatively small sample size, we were very thoughtful when choosing animals for research. The selected individuals represented different sire lines and different breeding farms, and unveiled the low amount of Holstein ancestral components (S3 Fig). Besides, the small sample sizes appear to be sufficient to obtain the reliable results using high-density SNP markers [56].

We found that 9.36% of variability was attributed to genetic differences between breeds (*F*_ST_ = 0.094), and the remaining 90.64% was the result of allelic variation within breeds. We observed slightly higher level of genetic variability in Yaroslavl (_U_*H*_E_ = 0.349, *A*_R_ = 1.967) and Kholmogor (_U_*H*_E_ = 0.361, *A*_R_ = 1.977) breeds (Table 1) than was previously detected using medium-density DNA BeadChip [9, 10]. Variability was significantly lower in the Yaroslavl breed than in the Kholmogor breed (*p* < 0.001), probably because of more drastic decline in the census population size. During the last 60 years, the number of Yaroslavl cattle has decreased by practically 20 times compared to that of Kholmogor breed, which decreased by 4.5 times [12]. Our data agreed with the findings of Xu et al. [15], who performed the genotyping of three commercial taurine breeds by using high-density DNA array and observed similar level of genetic variability (*H*_E_ = 0.337–0.356). In contrast, Stronen et al. [17] detected lower genetic variability in taurine breeds (*H*_E_ = 0.226 and 0.298); this was probably because of the small population size of the studied native breeds. We observed slight excess of heterozygotes in both the Russian breeds (_U_*F*_IS_ = -0.022 and -0.013 for Kholmogor and Yaroslavl breeds, respectively), which agrees with the findings obtained using DNA chips of lower density [9, 10]. The detected inbreeding coefficient in Holsteins (_U_*F*_IS_ = -0.010) was similar or lower than one which was discovered in recent reports (*F*_IS_ = −0.004 [60], *F*_IS_ = 0.0526 [15]), which might reflect the differences in sample origins and population sizes.

The *N*_E_ showed an overall decline for the studied breeds over generations (Fig 1A, S2 Fig). The most recent *N*_E_ values observed for Yaroslavl (*N*_E5_ = 111) and Kholmogor (*N*_E5_ = 130) cattle were higher than those in Holsteins (*N*_E5_ = 95) and exceeded the critical threshold of *N*_E_ = 100 estimated for long-term viability of discrete livestock breeds [61]. We observed four periods of accelerated decline in *N*_E_ during the last 45 generations (Fig 1B). The more ancient periods were observed between 28 and 23 as well as 18 and 15 generations ago, which probably reflect the World wars crisis. The subsequent acceleration was detected between 8 and 7 generations ago, which was most likely the result of the implementation of artificial insemination, which is associated with the decrease in bull number. The recent accelerated decline in *N*_E_ was found between 5 and 4 generations ago for Kholmogor breed and between 6 and 5 generations ago for Yaroslavl breed, when the *N*_E_ decreased from 130 to 115 and 113 to 111, respectively. The possible explanation for this could be the large decrease in the population size of the purebred cattle from the mid-1990s to the mid-2000s owing to the general decline of industry in the first decade of the post-Soviet period.

We observed visible differences in the autozygosity level among the studied breeds. The average ROH length (258.3 Mb) in Yaroslavl breed was similar to that in Holsteins (270.1 Mb) and exceeded that in the Kholmogor breed (147.6 Mb). The higher total ROH length in Yaroslavl breed might be attributed to the higher inbreeding owing to the lower population size. Among the different length classes, short ROH segments (1–2 Mb) were prevalent (Fig 2, S1 Table), which agreed with the findings obtained in different cattle populations [48, 62, 63].

The results of the PCA plot, Neighbor-Net analysis, and admixture clustering (S3 Fig) suggest a low contribution of Holstein ancestors (probably Holland cattle) in the formation of the architecture of the Yaroslavl and Kholmogor breeds, which has been proposed by several researchers [5, 9–11].

### Identification of genomic regions under putative selection in the Kholmogor and Yaroslavl breeds

We applied three different statistical methods (selection of top 0.1% SNPs by *F*_ST_ value for pair-wise breed comparison, identification of ROH islands shared by more than 50% of animals within each breed, and hapFLK analysis) to identify the genomic regions and genes that are affected by selection (S2 and S6 Tables). Considering a possible impact of genetic drift or recent inbreeding on ROHs [64], we selected the ROH islands to be under selection pressure, if they were confirmed by at least one more method or were observed in more than 70% of animals. Results obtained using multiple analytical approaches typically have higher informative power, furthermore, methods based on different approaches may also complement each other [20, 65]. Fifteen of seventeen regions, identified in Holsteins were overlapped with genomic regions, which were described by Yurchenko et al. [34] and eleven were overlapped with regions, which were validated by meta-assembly of selection signatures (S8 Table) [57]. We confirmed nine regions under putative selection in the genome of Yaroslavl cattle and six regions in the genome of Kholmogor cattle, which were previously identified by Yurchenko et al. [34] by using DCMS analysis [56] of medium-density SNP genotypes; in this study, the flanking positions of most of these regions were expanded (Table 3). In addition, we detected three new putative genomic regions affected by selection by using at least two different methods in the genome of Yaroslavl (BTA 4 at 4.74–5.36 Mbp, BTA 15 at 17.80–18.77 Mbp, and BTA 17 at 45.59–45.61 Mbp) and Kholmogor (BTA 12 at 82.40–81.69 Mbp, BTA 15 at 16.04–16.62 Mbp, and BTA 18 at 0.19–1.46 Mbp) breeds. Only two of the selected regions (localised on BTA 14 at 24.4–25.1 Mbp and BTA 16 at 42.5–43.5 Mb) overlapped in Yaroslavl, Kholmogor, and Holstein breeds. An additional overlapping region with Holsteins (localised on BTA 13 at 5.0–6.8 Mbp) was found in the Yaroslavl breed. Seventeen of twenty-one regions, found in the Yaroslavl and Kholmogor breeds were overlapped with the signature of selections, which were identified in other European breeds by meta-assembly [57], while two genomic regions in each breed were novel (S8 Table).

### Functional annotation of candidate genes localised within the selected regions

We performed functional annotation of genes localised within the putative regions carrying signals of selection in the genome of the studied breeds. Along with genes, which were previously described for Yaroslavl and Kholmogor cattle [34], we expanded the list of candidate genes by elucidating the known genomic regions and detecting additional selection sweeps in the genome of the studied breeds (Table 3, S6 Table).

We found two regions overlapping with the Holstein genomic region under selection pressure in both Yaroslavl and Kholmogor breeds (Table 3, S6 Table). One of these regions is localized on BTA 14 and contains the *XKR4*, *TMEM68*, *TGS1*, *LYN*, *RPS20*, *MOS*, *PLAG1*, and *CHCHD7* genes which are well-known candidates for growth, carcass-related traits [66–68], and feed intake [69–71]. The *PLAG1* gene (or *PLAG1-CHCHD7* region) is a strong candidate responsible for stature; body size, including height [72]; and weight in many cattle breeds [73–78]. Fink et al. [79] found a strong association of the *PLAG1* genotype with milk fat and protein yield. Utsunomiya et al. [80] provided an insightful account of the effect of *PLAG1* in the history of domesticated cattle. The polymorphic SNP BovineHD1400007259, located within the intronic region of the *PLAG1* gene, is considered as a causal mutation responsible for stature [81–83]. This mutation was segregated in both Yaroslavl (p_A_ = 0.367, q_C_ = 0.633; *H*_O_ = 0.323) and Kholmogor (p_A_ = 0.140, q_C_ = 0.860; *H*_O_ = 0.200) breeds, which could have resulted from selection in both breeds for animals of the larger size. Selection pressure on genes, involved in growth regulation is confirmed by identification of additional notable candidate genes with known effect on body size and/or carcass traits, including *Grb10* on BTA 4 [84–86], *PDGFRA*, *MAD2L* and *UBE2K* on BTA 6 [87–90], *SIRT6* on BTA 7 [91–93], and *ACAT1*, *KDELC2* on BTA 15 [94–96] in Yaroslavl breed, and *EGR1*, *HSPA9* on BTA 7 [97, 98], and *CDH15*, *DPEP1*, *GAS8*, *GALNS*, and *MC1R* on BTA 18 [30, 99–102] in Kholmogor breed. The second overlapping genomic region under positive selection in Yaroslavl, Kholmogor and Holstein breeds containing *TNFRSF1B*, *MFN2*, *PLOD1*, *KIAA2013*, *NPPB*, *NPPA*, *CLCN6*, *MTHFR*, *AGTRAP*, *FBXO2*, *MTOR*, *ANGPTL7*, *EXOSC10*, and *MASP2* genes was found on BTA16 (Table 3, S6 Table). The selected candidate genes are involved in different biological processes in cattle, including milk production—*MTHFR* [103] and *mTOR* genes [104, 105]; reproduction*—PLOD1* [106], *NPPA* and *NPPB* [107], *EXOSC10* genes [108]; immunity*—TNFRSF1B* [109] and *MASP2* genes [110, 111]; and metabolism—*MFN2* gene [112].

Additional large set of candidate genes (*LRRC24*, *LRRC14*, *RECQL4*, *HEATR7A*, *MFSD3*, *GPT*, *PPP1R16A*, *FOXH1*, *CYHR1*, *SLC39A4*, *CPSF1*, *ADCK5*, *FBXL6*, *BOP1*, *SCRT1*, *DGAT1*, *GPAA1*, *EXOSC4*, *PARP10*, *HSF1*, *OPLAH*, and *GRINA*), associated with milk production traits in Kholmogor cattle was found on BTA 14. These genes were shown to be involved in regulation of milk fatty acid composition [113–116], milk yield [117, 118], milk fat yield, and milk fat percentage [119–123]. In contrast to Kholmogor cattle, we identified only two candidate genes on BTA 5, which were associated with milk production traits in Yaroslavl cattle, including *TMCC3* and *DDX10* [124, 125]. The possible explanation for the presence of numerous milk trait genes affected by selection in Kholmogor cattle could be that one of the breeding targets for this breed from the middle of the 19^th^ century was to supply the Petersburg and Moscow citizens with high-quality dairy products [5]. This stimulated the selection of high-producing cows with excellent milk quality, suitable for butter and cheese production, and thus led to the alterations in the allele frequencies of genes which are involved in the regulation of milk production and composition.

We identified a few more candidate genes associated with reproduction traits, including *GSK3B* on BTA 1 [126], *ADAD1* on BTA 17 [127, 128], and *UBE3B* on BTA 21 [129, 130], in the Yaroslavl breed. These genes are associated mainly with female fertility traits, that can reflect the unique capacity of cows of Yaroslavl breed to produce healthy calves even with severe weight losses because of poor feeding in winter [4]. The genes detected in Kholmogor breed, including *WDR19* on BTA 6 [131], *NME5* on BTA 7 [132, 133], *NT5E* on BTA 9 [134], *PIWIL4* on BTA 15 [135], *TUBB3* and *UQCRFS1* on BTA 18 [136, 137], are mainly related to male fertility. Since the Kholmogor breed had become the breed of choice for the growing market of Petersburg and Moscow in the 19^th^ century, the use of bulls with increased fertility was preferred.

We detected selection pressure on genes involved in lipid metabolism in Yaroslavl cattle, including *PLIN4*, *PLIN5*, and *CREB3L3* on BTA 7 [138–141], *MFN2* on BTA 16 [112], and *NDUFV2* on BTA 24 [142]. This observation is probably associated with the capacity of cattle population of Central Russia to accumulate sufficient subcutaneous fat during the pasture period that helped them to survive under poor nutrition conditions in winter and to maintain pregnancy [4].

In Yaroslavl breed, we found a putative signature of selection around the *KIT* gene on BTA 6, which is a functional candidate for coat coloration in cattle, including white spotting patterns in the Holstein [143] and Fleckvieh cattle breeds [144]. According to the QTL database [58], the region on BTA 6 between 71.4 and 72.1 Mbp, where the *KIT* gene is localised, contains the genes associated with eye area pigmentation in Fleckvieh cattle [145]. The black pigmentation of the eye area is one of the well-known distinguishing features of Yaroslavl cattle (S1 Fig).

In summary, our study provides deeper insight into the genomic architecture of Yaroslavl and Kholmogor cattle breeds and allowed the identification of the genomic regions and genes that were affected by selection during the century-long history of breed formation. Our research results will be useful for the sustainable development and conservation of these two oldest Russian native cattle breeds.

## Supporting information

S1 Fig The photos of the pure-bred Yaroslavl and Kholmogor bulls. (TIF)

S2 Fig The effective population size (*N_E_*) across generations from approximately 500 generations ago based on linkage disequilibrium (LD) calculations. Note: Breeds: YRSL, Yaroslavl; KHLM, Kholmogor; HOL, Holsteins.
(TIF)

S3 Fig (A). Principal Component Analysis (PCA) plot showing the distribution of the studied cattle breeds in the dimensions of two coordinates. (B). A Neighbor-Net tree constructed on the basis of pairwise nucleotide genetic distances among individuals of the three studied breeds. (C). Admixture plot represents the cluster structure of the studied breeds for the number of clusters (*K*) 2 and 3. Different colours correspond to different ancestral populations. (D). Cross-validation (CV) error for the different number of clusters. Note: Axis X, first principal component (PC1); Axis Y, second principal component (PC2). The part of total genetic variability, which can be explained by each of the two components are indicated in curly parentheses. Breed: YRSL, Yaroslavl; KHLM, Kholmogor; HOL, Holsteins.
(TIF)

S4 Fig Distribution of ROHs within chromosomes. (a). BTA5, (b). BTA6, (c). BTA7, (d). BTA8, (e). BTA14, (f). BTA16, (g). BTA17, (h). BTA21. Note: Axis X, chromosomal positions (Mbps); Axis Y, the ratio of animals carrying ROHs (in percent); YRSL, Yaroslavl; KHLM, Kholmogor; HOL, Holsteins.
(TIF)

S1 Table Mean number and length of ROHs of different length classes. Note: Class: ROH length class (1–2 Mb, 2–4 Mb, 4–8 Mb, 8–16 Mb, and >16 Mb); breed: YRSL, Yaroslavl; KHLM, Kholmogor; HOL, Holsteins; n, number of animals; mean number of ROH: values, mean values for each length class and breed; sd, standard deviation; se, standard error; ci, confidence interval; %, ratio of the number of ROHs of certain length class in the common number of ROHs within each breed; mean length of ROH: values, mean values for each length class and breed; sd, standard deviation; se, standard error; ci, confidence interval; %, ratio of the length of ROHs of certain length class in the common length of ROHs within each breed.
(XLSX)

S2 Table Top 0.1% SNPs by *F_ST_* values during pair-wise comparison of breeds. Note: BTA, *Bos taurus* autosome; breed: YRSL, Yaroslavl; KHLM, Kholmogor; HOL, Holsteins; compared pairs of breeds indicated by different colour filling: KHLM/HOL, blue filling; YRSL/HOL, green filling; KHLM/YRSL, pink filling; SNP, SNPs selected in top 0.1% for each pair of breeds; POS, position of SNPs according to *Bos*_*taurus*_UMD_3.1.1 genome assembly (https://www.ncbi.nlm.nih.gov/assembly/GCF_000003055.6); NMISS, number of missing SNPs; *F*_ST_, *F*_ST_ value calculated for each SNP during pair-wise breed comparison; genes, genes within which or close (within window from 0.2 Mbp up-stream to 0.2 Mbp down-stream of the causal SNP) to which is localised the causal SNP; the candidate genes within which the causal SNPs are localised are indicted in bold.
(XLSX)

S3 Table Top 0.1% SNPs by *F_ST_* value, which are common for two pairs of breeds, during pair-wise breed comparison: YRSL/KHLM and YRSL/HOL, YRSL/HOL and KHLM/HOL, as well as KHLM/HOL and YRSL/KHLM. Note: breed: HOL, Holsteins; KHLM, Kholmogor; YRSL, Yaroslavl.
(XLSX)

S4 Table Putative selective sweeps identified in the hapFLK-based analysis. Note: BTA, *Bos taurus* autosome with the identified hapFLK regions; Breed: YRSL, Yaroslavl; KHLM, Kholmogor; HOL, Holsteins; Start and End positions, the positions of the first and last SNPs in the identified hapFLK region according to *Bos*_*taurus*_UMD_3.1.1 genome assembly (https://www.ncbi.nlm.nih.gov/assembly/GCF_000003055.6); Length, length of hapFLK regions (Mbp); No. of SNPs, the number of SNPs within localised hapFLK regions; the hapFLK regions are sorted by chromosome and then by start position on chromosome.
(XLSX)

S5 Table Genomic regions in Yaroslavl and Kholmogor cattle breeds under putative selection compared to those in Holsteins revealed by ROH analysis. Note: BTA, *Bos taurus* autosome with the overlapping ROH shared by more than 50% of the animals (the strongest ROH islands shared by more than 70% of animals are shown in bold); Breed: YRSL, Yaroslavl (indicated by green color); KHLM, Kholmogor (indicated by blue color); HOL, Holsteins (indicated by pink color); No. of SNPs, number of SNPs within an ROH island; Start and End positions, the positions of the first and last SNPs in an ROH island according to *Bos*_*taurus*_UMD_3.1.1 genome assembly (https://www.ncbi.nlm.nih.gov/assembly/GCF_000003055.6); Length, length of ROH island (Mb); Percent of animals, the percent of animals carrying the ROH island. The ROH islands are sorted by chromosome and then by start position on chromosome.
(XLSX)

S6 Table Genomic regions and genes affected by putative selection identified using HapFLK and ROH analyses, and overlapping regions identified by *F_ST_* calculations in Yaroslavl and Kholmogor cattle breeds. Note: BTA, *Bos taurus* autosome; Start_SNP and End_SNP, the names of the start and end SNPs on Bovine HD BeadChip (Illumina Inc., USA) flanking the genomic regions under putative selection; breed: YRSL, Yaroslavl; KHLM, Kholmogor; HOL, Holsteins; methods: *F*_*ST*_, top 0.1% SNPs by *F*_*ST*_ value during pair-wise breed comparison; *hapFLK*, regions identified by hapFLK analysis; *ROH_50%* and *ROH_70%*, ROH segments distributed in more than 50% and more than 70% of animals, respectively, within each of the studied breed (the ROH segments identified in more than 70% animals are shown in bold); nSNP, number of SNPs localised within the identified genomic region; Start position and End position, start and end positions of the genomic region affected by putative selection (Mbp) according to *Bos*_*taurus*_UMD_3.1.1 genome assembly (https://www.ncbi.nlm.nih.gov/assembly/GCF_000003055.6); length, the length of the identified genomic regions under putative selection (Mbp); No. of genes, number of genes localised within identified genomic regions; genes, the list of genes localised within identified genomic regions (genes localised within ROH segments identified in more than 70% of animals are shown in bold).
(XLSX)

S7 Table Selected genes under putative selection in Kholmogor and Yaroslavl cattle breeds. Note: ^1^Genes: *GSK3B*—glycogen synthase kinase 3 beta; *Grb10*—growth factor receptor-bound protein 10; *PLXNC1*—plexin C1; *TMCC3*—transmembrane and coiled-coil domain family 3; *FGD6*—FYVE, RhoGEF, and pH domain containing 6; *MAD2L1—*mitotic arrest deficient 2 like 1; *WDR19*—WD repeat domain 19; *KLB*—klotho beta; *RPL9*—ribosomal protein L9; *LIAS*—lipoic acid synthase; *UGDH*—UDP-glucose 6-dehydrogenase; UBE2K—ubiquitin-conjugating enzyme E2 K; *PDS5A*—PDS5 cohesin-associated factor A; *PDGFRA*—platelet-derived growth factor receptor, alpha polypeptide; KIT—V-kit Hardy–Zuckerman 4 feline sarcoma viral oncogene homolog; *TICAM1*—toll like receptor adaptor molecule 1; *PLIN5*—perilipin 5; *PLIN4*—perilipin 4; *SIRT6*—sirtuin 6; *CREB3L3*—cAMP-responsive element-binding protein 3 like 3; *NME5*—NME/NM23 family member 5; *EGR1*—early growth response 1; *HSPA9*—heat shock protein family A (Hsp70) member 9; *NT5E*—ecto-5′-nucleotidase; *LRRC24*—leucine-rich repeat-containing protein 24; *LRRC14*—leucine-rich repeat-containing protein 14; *RECQL4*—RecQ like helicase 4; *MFSD3*—major facilitator superfamily domain containing 3; *GPT*—glutamic-pyruvic transaminase; *PPP1R16A*—protein phosphatase 1 regulatory subunit 16A; *FOXH1*—forkhead box H1; *CYHR1*—cysteine and histidine rich 1; *SLC39A4*—solute carrier family 39 member 4; *CPSF1*—cleavage and polyadenylation specific factor 1; *ADCK5*—aarF domain-containing kinase 5; *FBXL6*—F-Box and leucine rich repeat protein 6; *SCRT1*—Scratch family transcriptional repressor 1; *DGAT1*—diacylglycerol acyltransferase 1; *HSF1*—Heat shock factor 1; *BOP1*—BOP1 ribosomal biogenesis factor; *HEATR7A*—maestro heat like repeat family member 1; *GPAA1*—glycosylphosphatidylinositol anchor attachment 1; *EXOSC4*—exosome component 4; *OPLAH*—5-oxoprolinase, ATP-hydrolysing; *GRINA*—glutamate ionotropic receptor NMDA type subunit-associated protein 1; *PARP10*—poly(ADP-ribose) polymerase family member 10; *XKR4*—XK, Kell blood group complex subunit-related family, member 4; *TMEM68*—transmembrane protein 8B; *TGS1*—trimethylguanosine synthase 1; *LYN*—LYN proto-oncogene, Src family tyrosine kinase; *RPS20*—ribosomal protein S20; *MOS*—MOS proto-oncogene, serine/threonine kinase; *PLAG1*—pleomorphic adenoma gene 1; *CHCHD7*—coiled-coil-helix-coiled-coil-helix domain containing 7; *PIWIL4*—piwi like RNA-mediated gene silencing 4; *GUCY1A2*—guanylate cyclase 1 soluble subunit alpha 2; *ACAT1*—acetyl-CoA acetyltransferase 1; *KDELC2*—KDEL (Lys-Asp-Glu-Leu) containing 2; *EXPH5*—exophilin 5; *DDX10*—DEAD-box helicase 10; *NPPB*—natriuretic peptide B; *NPPA*—natriuretic peptide A; *CLCN6*—chloride voltage-gated channel 6; *MTHFR*—methylenetetrahydrofolate reductase; *AGTRAP*—angiotensin II receptor associated protein; *FBXO2*—F-box protein 2; *TNFRSF1B*—tumour necrosis factor receptor superfamily, member 1B; *MFN2*—mitofusin 2; *PLOD1*—procollagen-lysine, 2-oxoglutarate 5-dioxygenase 1; *mTOR*—mammalian target of rapamycin; *ANGPTL7*—angiopoietin like 7; *EXOSC10*—exosome component 10; *MASP2*—mannose-binding lectin-associated serine protease 2; *IL2*—interleukin 2; *ADAD1*—adenosine deaminase domain containing 1; *UQCRFS1*—ubiquinol-cytochrome c reductase, Rieske iron–sulphur polypeptide 1; *GALNS*—galactosamine(*N*-acetyl)-6-sulfatesulfatase; *CBFA2T3*—CBFA2/RUNX1 partner transcriptional co-repressor 3; *CDH15*—cadherin15, type1, M-cadherin; *SPG7*—SPG7 matrix AAA peptidase subunit, paraplegin; *CPNE7*—copine 7; *DPEP1*—dipeptidase 1; *CHMP1A*—charged multivesicular body protein 1A; *CDK10*—cyclin dependent kinase 10; *SPATA2L*—spermatogenesis associated 2 like; *FANCA—*Fanconi anaemia, complementation group A; *TCF25*—transcription factor 25; *SPIRE2*—spire type actin nucleation factor 2; *TUBB3*—tubulin beta 3 class III; *GAS8*—growth arrest specific 8; *MC1R*—melanocortin 1 receptor; *UBE3A*—ubiquitin protein ligase E3A; *NDUFV2*—NADH dehydrogenase flavoprotein 2; breed: YRSL—Yaroslavl, KHLM—Kholmogor; BTA—*Bos taurus* autosome; positions—start and end positions of a gene (bp) according to *Bos*_*taurus*_UMD_3.1.1 genome assembly (https://www.ncbi.nlm.nih.gov/assembly/GCF_000003055.6).
(XLSX)

S8 Table Comparison of the identified genomic regions under putative selection with those identified by meta-assembly of selection signatures. Note: BTA, *Bos taurus* autosome; Breed: YRSL, Yaroslavl; KHLM, Kholmogor; HOL, Holsteins; genomic regions: start and end positions (Mbp) according to *Bos*_*taurus*_UMD_3.1.1 genome assembly (https://www.ncbi.nlm.nih.gov/assembly/GCF_000003055.6); methods used for the identification of the signature of selection: *F*_*ST*_, top 0.1% SNPs by *F*_ST_ value at pair-wise breed comparison; *hapFLK*, regions identified by hapFLK analysis; *ROH50*, ROH segments distributed in more than 50% of animals within each of the studied breed; ^1^present study; ^2^Yurchenko et al. [34]; ^3^meta-assembly of selection signatures, performed by Randhawa et al. [58]: overlapped MSS region (Mb)–overlapped genomic regions, which were validated by meta-selection scores; pairs of breeds, used for *F*_ST_ calculations: ^a^KHLM/HOL, ^b^YRSL/HOL, ^c^YRSL/KHLM; ^d^weak selection signal was also observed in Kholmogor breed.
(XLSX)

## References

[1] Scherf BD, Pilling D. The second report on the state of the world’s animal genetic resources for food and agriculture. Rome: FAO Commission on genetic resources for food and agriculture assessments; 2015 [Cited 2020 April 14]. Available from: http://www.fao.org/3/a-i4787e/index.html

[2] Dmitriev NG, Ernst LK. Animal genetic resources of the USSR. Rome: FAO and UNEP; 1989.

[3] Liskun EF. Russkie otrod’ya krupno-rogatogo skota. Moskva: Novyj agronom; 1928. Russian.

[4] Liskun EF. What is good about Russian Northern cattle. Petrograd: Publishing house of the people's Commissariat of agriculture; 1919 [Cited 2020 April 22]. Available from: https://www.booksite.ru/fulltext/liskun1/text.pdf.

[5] Liskun EF. Otechestvennye porody krupnogo rogatogo skota. Moskva: GISL; 1949. Russian.

[6] Diomidov AM, Zhirkovich EF. Razvedenie i porodv krupnogo rogatogo skota. Moskva-Leningrad: GIKSL; 1934. Russian.

[7] Felius M. Cattle breeds—an encyclopedia. Doetinchem: Senefelder Mis- set; 1995.

[8] Zinovieva NA, Dotsev AV, Sermyagin AA, Wimmers K, Reyer H, Sölkner J, et al Study of genetic diversity and population structure of five Russian cattle breeds using whole-genome SNP analysis. Sel'skokhozyaistvennaya Biologiya **[Agricultural Biology].** 2016; 51 (2): 788–800. 10.15389/agrobiology.2016.6.788eng

[9] Sermyagin AA, Dotsev AV, Gladyr EA, Traspov AA, Deniskova TE, Kostjunina OV, et al Whole-genome SNP analysis elucidates the genetic structure of Russian cattle and its relationship with Eurasian taurine breeds. Genetics, Selection, Evolution. 2018; 50: 37 10.1186/s12711-018-0408-8 29996786 PMC6042431

[10] Yurchenko A, Yudin N, Aitnazarov R, Plyusnina A, Brukhin V, Soloshenko V, et al Genome-wide genotyping uncovers genetic profiles and history of the Russian cattle breeds. Heredity (Edinb). 2018; 120:125–137. 10.1038/s41437-017-0024-3 29217829 PMC5837115

[11] Abdelmanova AS, Kharzinova VR, Volkova VV, Mishina AI, Dotsev AV, Sermyagin AA, et al Genetic Diversity of Historical and Modern Populations of Russian Cattle Breeds Revealed by Microsatellite Analysis. Genes. 2020; 11: 940 10.3390/genes11080940 32824045 PMC7463645

[12] Zinovieva NA, Sermyagin AA, Dotsev AV, Boronetslaya OI, Petrikeeva LV, Abdelmanova AS, et al Animal genetic resources: developing the research of allele pool of Russian cattle breeds—minireview. Sel’skokhozyaistvennaya Biologiya [Agricultural Biology]. 2019; 54 (4): 631–641. 10.15389/agrobiology.2019.4.631eng

[13] Li MH, Kantanen J. Genetic structure of Eurasian cattle (Bos taurus) based on microsatellites: clarification for their breed classification. Anim Genet. 2010; 41: 150–158. 10.1111/j.1365-2052.2009.01980.x 19845598

[14] Dotsev AV, Sermyagin AA, Shakhin AV, Paronyan IA, Plemyashov KV, Reyer H, et al Evaluation of current gene pool of Kholmogor and Black-and-white cattle breeds based on whole genome SNP analysis. Vavilov Journal of Genetics and Breeding. 2018; 22(6). 10.18699/VJ18.418

[15] Xu L, Bickhart DM, Cole JB, Schroeder SG, Song J, Van Tassell CP, et al Genomic Signatures Reveal New Evidences for Selection of Important Traits in Domestic Cattle. Mol Biol Evol. 2014; 32 (3): 711–725. 10.1093/molbev/msu333 25431480 PMC4441790

[16] Bahbahani H, Tijjani A, Mukasa C, Wragg D, Almathen F, Nash O, et al Signatures of Selection for Environmental Adaptation and Zebu × Taurine Hybrid Fitness in East African Shorthorn Zebu. Frontiers in Genetics. 2017; 8: 8:68. 10.3389/fgene.2017.00008 28642786 PMC5462927

[17] Stronen AV, Pertoldi C, Iacolina L, Kadarmideen HN, Kristensen TN. Genomic analyses suggest adaptive differentiation of northern European native cattle breeds. Evol Appl. 2019; 12: 1096–1113. 10.1111/eva.12783 31293626 PMC6597895

[18] Kijas JW, Lenstra JA, Hayes B, Boitard S, Neto LRP, Cristobal MS, et al Genome-wide analysis of the world’s sheep breeds reveals high levels of historic mixture and strong recent selection. PLoS Biol. 2012; 10: 1–14. 10.1371/journal.pbio.1001258 22346734 PMC3274507

[19] Qanbari S, Gianola D, Hayes B, Schenkel F, Miller S, Moore S, et al Application of site and haplotype-frequency based approaches for detecting selection signatures in cattle. BMC Genomics. 2011; 12:318 10.1186/1471-2164-12-318 21679429 PMC3146955

[20] Cadzow M, Boocock J, Nguyen HT, Wilcox P, Merriman TR, Black MA. A bioinformatics workflow for detecting signatures of selection in genomic data. Front Genet. 2014; 5: 293 10.3389/fgene.2014.00293 25206364 PMC4144660

[21] Wright S. The genetical structure of populations. Ann Eugen. 1951; 15: 323–354. 10.1111/j.1469-1809.1949.tb02451.x 24540312

[22] Holsinger KE, Weir BS. Genetics in geographically structured populations: defining, estimating and interpreting F(ST). Nat. Rev. Genet. 2009; 10: 639–650. 10.1038/nrg2611 19687804 PMC4687486

[23] Akey JM, Zhang G, Zhang K, Jin L, Shriver MD. Interrogating a high-density SNP map for signatures of natural selection. Genome Res. 2002; 12(12):1805–14. 10.1101/gr.631202 12466284 PMC187574

[24] Zhao F, McParland S, Kearney F, Du L, Berry DP. Detection of selection signatures in dairy and beef cattle using high-density genomic information. Genet Sel Evol. 2015; 47:49 10.1186/s12711-015-0127-3 26089079 PMC4472243

[25] Fariello MI, Boitard S, Naya H, SanCristobal M, Servin B Detecting signatures of selection through haplotype differentiation among hierarchically structured populations. Genetics. 2013; 193 (3): 929–41. 10.1534/genetics.112.147231 23307896 PMC3584007

[26] Ferenčaković M, Hamzić E, Gredler B, Solberg TR, Klemetsdal G, Curik I, et al Estimates of autozygosity derived from runs of homozygosity: empirical evidence from selected cattle populations. J Anim Breed Genet. 2013; 130: 286–93. 10.1111/jbg.12012 23855630

[27] Peripolli E, Munari DP, Silva MVGB, Lima ALF, Irgang R, Baldi F. Runs of homozygosity: current knowledge and applications in livestock. Animal Genetics. 2016; 48: 255–271. 10.1111/age.12526 27910110

[28] Pemberton TJ, Absher D, Feldman MW, Myers RM, Rosenberg NA, Li JZ. Genomic patterns of homozygosity in worldwide human populations. Am J Hum Genet. 2012; 91: 275–292. 10.1016/j.ajhg.2012.06.014 22883143 PMC3415543

[29] Maiorano AM, Lourenco DL, Tsuruta S, Ospina AMT, Stafuzza NB, Masuda Y, et al Assessing genetic architecture and signatures of selection of dual purpose Gir cattle populations using genomic information. Plos One. 2018; 13(8): e0200694 10.1371/journal.pone.0200694 30071036 PMC6071998

[30] Pintus E, Sorbolini S, Albera A, Gaspa G, Dimauro C, Steri R, et al Use of locally weighted scatterplot smoothing (LOWESS) regression to study selection signatures in Piedmontese and Italian Brown cattle breeds. Anim Genet. 2014; 45(1): 1–11. 10.1111/age.12076 23889699

[31] Pérez O'Brien AM, Utsunomiya YT, Mészáros G, Bickhart DM, Liu GE, Van Tassell CP, et al Assessing signatures of selection through variation in linkage disequilibrium between taurine and indicine cattle. Genet Sel Evol. 2014; 4 (46):19 10.1186/1297-9686-46-19 24592996 PMC4014805

[32] Peripolli E, Stafuzza NB, Munari DP, Lima ALF, Irgang R, Machado MA, et al Assessment of runs of homozygosity islands and estimates of genomic inbreeding in Gyr (Bos indicus) dairy cattle. BMC Genomics. 2018; 19: 34 10.1186/s12864-017-4365-3 29316879 PMC5759835

[33] Iso-Touru T, Tapio M, Vilkki J, Kiseleva T, Ammosov I, Ivanova Z, et al Genetic diversity and genomic signatures of selection among cattle breeds from Siberia, eastern and northern Europe. Anim Genet. 2016; 47 (6): 647–657. 10.1111/age.12473 27629771

[34] Yurchenko AA, Daetwyler HD, Yudin N, Schnabel RD, Vander Jagt CJ, et al Scans for signatures of selection in Russian cattle breed genomes reveal new candidate genes for environmental adaptation and acclimation. Sci Rep. 2018; 8(1): 12984 10.1038/s41598-018-31304-w 30154520 PMC6113280

[35] Bahbahani H, Tijjani A, Mukasa C, Wragg D, Almathen F, Nash O, et al Data from: Signatures of selection for environmental adaptation and zebu x taurine hybrid fitness in East African Shorthorn Zebu. Dryad. 2018; Dataset. 10.5061/dryad.38jp6 28642786 PMC5462927

[36] R Core Team. R: A language and environment for statistical computing. Vienna: R Foundation for Statistical Computing; 2018.

[37] Fan JB, Oliphant A, Shen R, Kermani BG, Garcia F, Gunderson KL, et al Highly parallel SNP genotyping. Cold Spring Harb Symp Quant Biol. 2003; 68: 69–78. 10.1101/sqb.2003.68.69 15338605

[38] Purcell S, Neale B, Todd-Brown K, Thomas L, Ferreira MAR, Bender D, et al PLINK: a tool set for whole-genome association and population-based linkage analyses. Am J Hum Genet. 2007; 81 (3): 559–575. 10.1086/519795 17701901 PMC1950838

[39] Nei M. Estimation of average heterozygosity and genetic distance from small number of individuals. Genetics. 1978; 89, 583–590. 17248844 10.1093/genetics/89.3.583 PMC1213855

[40] Keenan K, McGinnity P, Cross TF, Crozier WW, Prodohl PA. diveRsity: An R package for the estimation of population genetics parameters and their associated errors. Methods in Ecology and Evolution. 2013; 4: 782–788. 10.1111/2041-210X.12067

[41] Barbato M, Orozco-terWengel P, Tapio M, Bruford MW. SNeP: a tool to estimate trends in recent effective population size trajectories using genome-wide SNP data. Front Genet. 2015; 6:109 10.3389/fgene.2015.00109 25852748 PMC4367434

[42] Corbin LJ, Liu AY, Bishop SC, Woolliams JA. Estimation of historical effective population size using linkage disequilibria with marker data. J Anim Breed Genet. 2012; 129(4): 257–270. 10.1111/j.1439-0388.2012.01003.x 22775258

[43] Sved JA, Feldman MW. Correlation and probability methods for one and two loci. Theoretical population biology. 1973; 4(1):129–32. 10.1016/0040-5809(73)90008-7 4726005

[44] Marras G, Gaspa G, Sorbolini S, Dimauro C, Ajmone-Marsam P, Valentini A, et al Analysis of runs of homozygosity and their relationship with inbreeding in five cattle breeds farmed in Italy. Anim Genet. 2014; 46:110–121. 10.1111/age.12259 25530322

[45] Biscarini F, Paolo Cozzi P, Gaspa G, Marras G. detectRUNS: Detect Runs of homozygosity and runs of heterozygosity in diploid genomes. R package version 0.9.5. 2018; Retrieved from https://CRAN.R-project.org/package=detectRUNS

[46] Ferenčaković M, Sölkner J, Curik I. Estimating autozygosity from high-throughput information: effects of SNP density and genotyping errors. Genet Sel Evol. 2013; 45(1):42 10.1186/1297-9686-45-42 24168655 PMC4176748

[47] Lencz T, Lambert C, DeRosse P, Burdick KE, Morgan TV, Kane JM, et al Runs of homozygosity reveal highly penetrant recessive loci in schizophrenia. Proc Natl Acad Sci USA. 2007; 104(50): 19942–19947. 10.1073/pnas.0710021104 18077426 PMC2148402

[48] Purfield DC, Berry DP, McParland S, Bradley DG. Runs of homozygosity and population history in cattle. BMC Genet. 2012; 13:70 10.1186/1471-2156-13-70 22888858 PMC3502433

[49] Wickham H. ggplot2: Elegant graphics for data analysis. Springer-Verlag, NY, 2009.

[50] Huson DH, Bryant D. Application of phylogenetic networks in evolutionary studies. Molecular Biology and Evolution, 2006; 23(2):254–67. 10.1093/molbev/msj030 16221896

[51] Alexander DH, Novembre J, Lange K. Fast model-based estimation of ancestry in unrelated individuals. Genome Res. 2009; 19: 1655–1664. 10.1101/gr.094052.109 19648217 PMC2752134

[52] Francis RM. POPHELPER: An R package and web app to analyse and visualize population structure. Mol Ecol Resour. 2017; 17: 27–32. 10.1111/1755-0998.12509 26850166

[53] Weir BS, Cockerham CC. Estimating F-statistics for the analysis of population structure. Evolution. 1984; 38: 1358–1370. 10.1111/j.1558-5646.1984.tb05657.x 28563791

[54] Scheet P, Stephens M. A fast and flexible statistical model for large-scale population genotype data: applications to inferring missing genotypes and haplotypic phase. Am J Hum Genet. 2006; 78:629–644. 10.1086/502802 16532393 PMC1424677

[55] Storey JD, Bass AJ, Dabney A, Robinson D. qvalue: Q-value estimation for false discovery rate control. R package version 2.18.0. 2019; Retrieved from http://github.com/jdstorey/qvalue

[56] Ma Y, Ding X, Qanbari S, Weigend S, Zhang Q, Simianer H. Properties of different selection signature statistics and a new strategy for combining them. Heredity. 2015; 115(5): 426–436. 10.1038/hdy.2015.42 25990878 PMC4611237

[57] Randhawa IAS, Khatkar MS, Thomson PC, Raadsma HW. A Meta-assembly of selection signatures in cattle. Plos One. 2016; 11(4): e0153013 10.1371/journal.pone.0153013 27045296 PMC4821596

[58] Hu Z-L, Park CA, Reecy JM. Building a livestock genetic and genomic information knowledgebase through integrative developments of Animal QTLdb and CorrDB. Nucleic Acids Research. 2019; 47: 701–710. 10.1093/nar/gky1084 30407520 PMC6323967

[59] Hoffmann I. Adaptation to climate change–exploring the potential of locally adapted breeds. Animal. 2013; 7, 346–362. 10.1017/S1751731113000815 23739476

[60] Huson HJ, Sonstegard TS, Godfrey J, Hambrook D, Wolfe C, Wiggans G, et al A Genetic investigation of island jersey cattle, the foundation of the jersey breed: comparing population structure and selection to Guernsey, Holstein, and United States jersey cattle. Front Genet. 2020; 11, 366 10.3389/fgene.2020.00366 32362912 PMC7181675

[61] Meuwissen T. Genetic management of small populations: a review. Acta Agric Scandinavica, Section A—Anim. Sci. 2009; 59: 71–79. 10.1080/09064700903118148

[62] Addo S, Klingel S, Hinrichs D, Thaller G Runs of Homozygosity and NetView analyses provide new insight into the genome-wide diversity and admixture of three German cattle breeds. Plos One. 2019; 14(12): e0225847 10.1371/journal.pone.0225847 31800604 PMC6892555

[63] Xu L, Zhao G, Yang L, Zhu B, Chen Y, et al Genomic patterns of homozygosity in Chinese local cattle. Sci Rep. 2019; 9: 16977 10.1038/s41598-019-53274-3 31740716 PMC6861314

[64] Kim ES, Cole JB, Huson H, Wiggans GR, Van Tassell CP, Crooker BA, et al Effect of artificial selection on runs of homozygosity in U.S. Holstein cattle. Plos One. 2013; 8(11): e80813 10.1371/journal.pone.0080813 24348915 PMC3858116

[65] François O, Martins H, Caye K, Schoville SD. Controlling false discoveries in genome scans for selection. Molecular Ecology. 2016; 25 (2): 454–469. 10.1111/mec.13513 26671840

[66] Cheruiyot EK, Bett RC, Amimo JO, Zhang Y, Mrode R, Mujibi FDN. Signatures of selection in admixed dairy cattle in Tanzania. Front Genet. 2018; 9:607 10.3389/fgene.2018.00607 30619449 PMC6305962

[67] Taye M, Yoon J, Dessie T, Cho S, Oh SJ, Lee HK, et al Deciphering signature of selection affecting beef quality traits in Angus cattle. Genes Genomics. 2018; 40(1): 63–75. 10.1007/s13258-017-0610-z 29892901

[68] Grigoletto L, Ferraz JBS, Oliveira HR, Eler JP, Bussiman FO, Abreu Silva BC, et al Genetic architecture of carcass and meat quality traits in Montana tropical composite beef cattle. Front Genet. 2020; 11:123 10.3389/fgene.2020.00123 32180796 PMC7057717

[69] Lindholm-Perry AK, Kuehn LA, Smith TP, Ferrell CL, Jenkins TG, Freetly HC, et al A region on BTA14 that includes the positional candidate genes LYPLA1, XKR4 AND TMEM68 is associated with feed intake and growth phenotypes in cattle. Anim Genet. 2012; 43: 216–219. 10.1111/j.1365-2052.2011.02232.x 22404358

[70] Pryce JE, Arias J, Bowman PJ, Davis SR, Macdonald KA, Waghorn GC, et al Accuracy of genomic predictions of residual feed intake and 250-day body weight in growing heifers using 625,000 single nucleotide polymorphism markers. J Dairy Sci. 2012; 95(4): 2108–2119. 10.3168/jds.2011-4628 22459856

[71] de Las Heras-Saldana S, Clark SA, Duijvesteijn N, Gondro C, van der Werf JHJ, Chen Y. Combining information from genome-wide association and multi-tissue gene expression studies to elucidate factors underlying genetic variation for residual feed intake in Australian Angus cattle. BMC Genomics. 2019; 20(1): 939 10.1186/s12864-019-6270-4 31810463 PMC6898931

[72] Zhong JL, Xu JW, Wang J, Wen YF, Niu H, Zheng L, et al A novel SNP of PLAG1 gene and its association with growth traits in Chinese cattle. Gene. 2019; 689: 166–171. 10.1016/j.gene.2018.12.018 30580072

[73] Littlejohn M, Grala T, Sanders K, Walker C, Waghorn G, Macdonald K, et al Genetic variation in PLAG1 associates with early life body weight and peripubertal weight and growth in Bos taurus. Anim Genet. 2012; 43 (5): 591–594. 10.1111/j.1365-2052.2011.02293.x 22497486

[74] Nishimura S, Watanabe T, Mizoshita K, Tatsuda K, Fujita T, Watanabe N, et al Genome-wide association study identified three major QTL for carcass weight including the PLAG1-CHCHD7 QTN for stature in Japanese Black cattle. BMC Genet. 2012; 13: 40 10.1186/1471-2156-13-40 22607022 PMC3403917

[75] Li Z, Wu M, Zhao H, Fan L, Zhang Y, Yuan T, et al The PLAG1 mRNA expression analysis among genetic variants and relevance to growth traits in Chinese cattle. Anim Biotechnol. 2019 6 28:1–8. 10.1080/10495398.2019.1632207 31253059

[76] An B, Xia J, Chang T, Wang X, Xu L, Zhang L, et al Genome-wide association study reveals candidate genes associated with body measurement traits in Chinese Wagyu beef cattle. Anim Genet. 2019; 50 (4): 386–390. 10.1111/age.12805 2019. 31179577

[77] Kim HJ, Sharma A, Lee SH, Lee DH, Lim DJ, Cho YM, et al Genetic association of PLAG1, SCD, CYP7B1 and FASN SNPs and their effects on carcass weight, intramuscular fat and fatty acid composition in Hanwoo steers (Korean cattle). Anim. Genet. 2017; 48 (2): 251–252. 10.1111/age.12523 27878829

[78] Smith JL, Wilson ML, Nilson SM, Rowan TN, Oldeschulte DL, Schnabel RD, et al Genome-wide association and genotype by environment interactions for growth traits in U.S. Gelbvieh cattle. BMC Genomics. 2019; 20 (1): 926 10.1186/s12864-019-6231-y 31801456 PMC6892214

[79] Fink T, Tiplady K, Lopdell T, Johnson T, Snell RG, Spelman RJ, et al Functional confirmation of PLAG1 as the candidate causative gene underlying major pleiotropic effects on body weight and milk characteristics. Sci Rep. 2017; 7: 44793 10.1038/srep44793 . 28322319 PMC5359603

[80] Utsunomiya YT, Carmo AS, Neves HH, Carvalheiro R, Matos MC, Zavarez LB, et al Genome-wide mapping of loci explaining variance in scrotal circumference in Nellore cattle. Plos One. 2014; 9(2):e88561 10.1371/journal.pone.0088561 24558400 PMC3928245

[81] Karim L, Takeda H, Lin L, Druet T, Arias JA, Baurain D, et al Variants modulating the expression of a chromosome domain encompassing PLAG1 influence bovine stature. Nat Genet. 2011; 43: 405–413. 10.1038/ng.814 21516082

[82] Bouwman AC, Daetwyler HD, Chamberlain AJ, Ponce CH, Sargolzaei M, Schenkel FS, et al Meta-analysis of genome-wide association studies for cattle stature identifies common genes that regulate body size in mammals. Nat. Genet. 2018; 50: 362 10.1038/s41588-018-0056-5 29459679

[83] Hou J, Qu K, Jia P, Hanif Q, Zhang J, Chen N, et al A SNP in PLAG1 is associated with body height trait in Chinese cattle. Animal Genetics. 2020; 51(1): 87–90. 10.1111/age.12872 31643102

[84] Magee DA, Sikora KM, Berkowicz EW, Berry DP, Howard DJ, Mullen MP, et al DNA sequence polymorphisms in a panel of eight candidate bovine imprinted genes and their association with performance traits in Irish Holstein-Friesian cattle. BMC Genet. 2010; 11: 93 10.1186/1471-2156-11-93 20942903 PMC2965127

[85] Imumorin IG, Kim EH, Lee YM, De Koning DJ, van Arendonk JA, De Donato M, et al Genome scan for parent-of-origin QTL effects on bovine growth and carcass traits. Front Genet. 2011; 2:44 10.3389/fgene.2011.00044 22303340 PMC3268597

[86] Xu L, Yang L, Wang L, Zhu B, Chen Y, Gao H, et al Probe-based association analysis identifies several deletions associated with average daily gain in beef cattle. BMC Genomics. 2019; 20 (1): 31 10.1186/s12864-018-5403-5 30630414 PMC6327516

[87] Taye M, Kim J, Yoon SH, Lee W, Hanotte O, Dessie T, et al Whole genome scan reveals the genetic signature of African Ankole cattle breed and potential for higher quality beef. BMC Genet. 2017; 18 (1): 11 10.1186/s12863-016-0467-1 28183280 PMC5301378

[88] Sadkowski T, Jank M, Oprzadek J, Motyl T. Age-dependent changes in bovine skeletal muscle transcriptomic profile. J Physiol Pharmacol. 2006; 57 (7): 95–110. 17228098

[89] Gomes RDC, Silva SDL, Carvalho ME, Rezende FMD, Pinto LFB, Santana MHDA, et al Protein synthesis and degradation gene SNPs related to feed intake, feed efficiency, growth, and ultrasound carcass traits in Nellore cattle. Genet Mol Res. 2013; 12 (3): 2923–2936. 10.4238/2013.August.12.8 24065648

[90] Srikanth K, Lee SH, Chung KY, Park JE, Jang GW, Park MR, et al A Gene-set enrichment and protein-protein interaction network-based GWAS with regulatory SNPs identifies candidate genes and pathways associated with carcass traits in Hanwoo cattle. Genes (Basel). 2020; 11 (3): E316 10.3390/genes11030316 32188084 PMC7140899

[91] Gui L, Jiang B, Zhang Y, Zan L. Sequence variants in the bovine silent information regulator 6, their linkage and their associations with body measurements and carcass quality traits in Qinchuan cattle. Gene. 2015; 559: 16–21. 10.1016/j.gene.2015.01.008 25576955

[92] Raza SHA, Khan R, Abdelnour SA, Abd El-Hack ME, Khafaga AF, Taha A, et al Advances of molecular markers and their application for body variables and carcass traits in Qinchuan cattle. Genes (Basel). 2019; 10 (9): 717 10.3390/genes10090717 31533236 PMC6771018

[93] Gui LS, Raza SHA, Garcia M, Sun YG, Ullah IR, Han YC. Genetic variants in the SIRT6 transcriptional regulatory region affect gene activity and carcass quality traits in indigenous Chinese beef cattle (Bos taurus). BMC Genom. 2018; 19: 785 10.1186/s12864-018-5149-0 30382814 PMC6211504

[94] Silva-Vignato B, Coutinho LL, Poleti MD, Cesar ASM, Moncau CT, Regitano LCA, et al Gene co-expression networks associated with carcass traits reveal new pathways for muscle and fat deposition in Nelore cattle. BMC Genomics. 2019; 20 (1): 32 10.1186/s12864-018-5345-y 30630417 PMC6329100

[95] Kern RJ, Zarek CM, Lindholm-Perry AK, Kuehn LA, Snelling WM, Freetly HC, et al Ruminal expression of the NQO1, RGS5, and ACAT1 genes may be indicators of feed efficiency in beef steers. Anim Genet. 2017; 48 (1): 90–92. 10.1111/age.12490 27611366

[96] Serão NV, González-Peña D, Beever JE, Bollero GA, Southey BR, Faulkner DB, et al Bivariate genome-wide association analysis of the growth and intake components of feed efficiency. PLoS One. 2013; 8 (10): e78530 10.1371/journal.pone.0078530 24205251 PMC3812149

[97] Huang W, Guo Y, Du W, Zhang X, Li A, Miao X. Global transcriptome analysis identifies differentially expressed genes related to lipid metabolism in Wagyu and Holstein cattle. Sci Rep. 2017; 7 (1): 5278 10.1038/s41598-017-05702-5 28706200 PMC5509646

[98] Picard B, Gagaoua M. Meta-proteomics for the discovery of protein biomarkers of beef tenderness: An overview of integrated studies. Food Res Int. 2020; 127: 108739 10.1016/j.foodres.2019.108739 31882086

[99] Foote AP, Keel BN, Zarek CM, Lindholm-Perry AK. Beef steers with average dry matter intake and divergent average daily gain have altered gene expression in the jejunum. J Anim Sci. 2017; 95 (10): 4430–4439. 10.2527/jas2017.1804 29108031

[100] Braz CU, Taylor JF, Bresolin T, Espigolan R, Feitosa FLB, Carvalheiro R, et al Sliding window haplotype approaches overcome single SNP analysis limitations in identifying genes for meat tenderness in Nelore cattle. BMC Genet. 2019; 20 (1): 8 10.1186/s12863-019-0713-4 30642245 PMC6332854

[101] Gutiérrez-Gil B, Arranz JJ, Wiener P. An interpretive review of selective sweep studies in Bos taurus cattle populations: identification of unique and shared selection signals across breeds. Front Genet. 2015; 6: 167 10.3389/fgene.2015.00167 26029239 PMC4429627

[102] McLean KL, Schmutz SM. Associations of melanocortin 1 receptor genotype with growth and carcass traits in beef cattle. Can J Anim Sci. 2009; 89: 295–300. 10.4141/CJAS08094

[103] Menzies KK, Lefèvre C, Macmillan KL, Nicholas KR. Insulin regulates milk protein synthesis at multiple levels in the bovine mammary gland. Funct Integr Genomics. 2009; 9 (2): 197–217. 10.1007/s10142-008-0103-x 19107532

[104] Luo C, Zhao S, Dai W, Zheng N, Wang J. Proteomic analysis of lysosomal membrane proteins in bovine mammary epithelial cells illuminates potential novel lysosome functions in lactation. J Agric Food Chem. 2018; 66 (49): 13041–13049. 10.1021/acs.jafc.8b04508 30499671

[105] Liu Y, Wang X, Zhen Z, Yu Y, Qiu Y, Xiang W. GRP78 regulates milk biosynthesis and the proliferation of bovine mammary epithelial cells through the mTOR signaling pathway. Cell Mol Biol Lett. 2019; 24: 57 10.1186/s11658-019-0181-x 31660059 PMC6805561

[106] Regassa A, Rings F, Hoelker M, Cinar U, Tholen E, Looft C, et al Transcriptome dynamics and molecular cross-talk between bovine oocyte and its companion cumulus cells. BMC Genomics. 2011; 12: 57 10.1186/1471-2164-12-57 21261964 PMC3045333

[107] De Cesaro MP, Macedo MP, Santos JT, Rosa PR, Ludke CA, Rissi VB, et al Natriuretic peptides stimulate oocyte meiotic resumption in bovine. Anim Reprod Sci. 2015; 159: 52–59. 10.1016/j.anireprosci.2015.05.012 26051611

[108] Jamin SP, Petit FG, Kervarrec C, Smagulova F, Illner D, Scherthan H, et al EXOSC10/Rrp6 is post-translationally regulated in male germ cells and controls the onset of spermatogenesis. Sci Rep. 2017; 7 (1): 15065 10.1038/s41598-017-14643-y 29118343 PMC5678167

[109] Wang XG, Ju ZH, Hou MH, Jiang Q, Yang CH, Zhang Y, et al Deciphering transcriptome and complex alternative splicing transcripts in mammary gland tissues from cows naturally infected with staphylococcus aureus mastitis. Plos One. 2016; 11 (7): e0159719 10.1371/journal.pone.0159719 27459697 PMC4961362

[110] Wu J, Bai JY, Li L, Huang S, Li CM, Wang GL. Genetic polymorphisms of the BMAP-28 and MASP-2 genes and their correlation with the somatic cell score in Chinese Holstein cattle. Genetics and Molecular Research: GMR. 2015; 14 (1): 1–8 10.4238/2015.January.15.1 25729929

[111] Zhang H, Wei Y, Zhang F, Liu Y, Li Y, Li G, et al Polymorphisms of MASP2 gene and its relationship with mastitis and milk production in Chinese Holstein cattle. Biotechnology & Biotechnological Equipment. 2019; 33 (1): 589–596. 10.1080/13102818.2019.1596755

[112] Dong J, Loor JJ, Zuo R, Chen X, Liang Y, Wang Y, et al Low abundance of mitofusin 2 in dairy cows with moderate fatty liver is associated with alterations in hepatic lipid metabolism. J Dairy Sci. 2019; 102 (8): 7536–7547. 10.3168/jds.2019-16544 31178189

[113] Sanchez MP, Govignon-Gion A, Croiseau P, Fritz S, Hozé C, Miranda G, et al Within-breed and multi-breed GWAS on imputed whole-genome sequence variants reveal candidate mutations affecting milk protein composition in dairy cattle. Genet Sel Evol. 2017; 49 (1): 68 10.1186/s12711-017-0344-z 28923017 PMC5604355

[114] Palombo V, Milanesi M, Sgorlon S, Capomaccio S, Mele M, Nicolazzi E, et al Genome-wide association study of milk fatty acid composition in Italian Simmental and Italian Holstein cows using single nucleotide polymorphism arrays. J Dairy Sci. 2018; 101 (12): 11004–11019. 10.3168/jds.2018-14413 30243637

[115] Do DN, Schenkel FS, Miglior F, Zhao X, Ibeagha-Awemu EM. Genome wide association study identifies novel potential candidate genes for bovine milk cholesterol content. Sci Rep. 2018; 8 (1): 13239 10.1038/s41598-018-31427-0 30185830 PMC6125589

[116] Zhou C, Li C, Cai W, Liu S, Yin H, Shi S, et al Genome-wide association study for milk protein composition traits in a Chinese Holstein population using a single-step approach. Front Genet. 2019; 10: 72 10.3389/fgene.2019.00072 30838020 PMC6389681

[117] Grisart B, Farnir F, Karim L, Cambisano N, Kim J, Kvasz A, et al Genetic and functional confirmation of the causality of the DGAT1 K232A quantitative trait nucleotide in affecting milk yield and composition. Proc Natl Acad Sci U S A. 2004; 101: 2398–403. 10.1073/pnas.0308518100 14983021 PMC356962

[118] Wang D, Ning C, Liu JF, Zhang Q, Jiang L. Short communication: Replication of genome-wide association studies for milk production traits in Chinese Holstein by an efficient rotated linear mixed model. J Dairy Sci. 2019; 102 (3): 2378–2383. 10.3168/jds.2018-15298 30639022

[119] Fontanesi L, Calò DG, Galimberti G, Negrini R, Marino R, Nardone A, et al A candidate gene association study for nine economically important traits in Italian Holstein cattle. Anim Genet. 2014; 45 (4): 576–80. 10.1111/age.12164 24796806

[120] Cochran SD, Cole JB, Null DJ, Hansen PJ. Discovery of single nucleotide polymorphisms in candidate genes associated with fertility and production traits in Holstein cattle. BMC Genet. 2013; 14: 49 10.1186/1471-2156-14-49 23759029 PMC3686577

[121] Nayeri S, Sargolzaei M, Abo-Ismail MK, May N, Miller SP, Schenkel F, et al Genome-wide association for milk production and female fertility traits in Canadian dairy Holstein cattle. BMC Genet. 2016; 17 (1): 75 10.1186/s12863-016-0386-1 27287773 PMC4901445

[122] Weller JI, Bickhart DM, Wiggans GR, Tooker ME, O’Connell JR, Jiang J, et al Determination of quantitative trait nucleotides by concordance analysis between quantitative trait loci and marker genotypes of US Holsteins. J Dairy Sci. 2018; 101: 9089–107. 10.3168/jds.2018-14816 30031583

[123] Ibeagha-Awemu EM, Peters SO, Akwanji KA, Imumorin IG, Zhao X. High density genome wide genotyping-by-sequencing and association identifies common and low frequency SNPs, and novel candidate genes influencing cow milk traits. Sci Rep. 2016; 6: 31109 10.1038/srep31109 27506634 PMC4979022

[124] Stella А, Ajmone-Marsan P, Lazzari B, Boettcher P. Identification of selection signatures in cattle breeds selected for dairy production. Genetics. 2010; 185 (4): 1451–1461. 10.1534/genetics.110.116111 20479146 PMC2927769

[125] Zhu B, Niu H, Zhang W, Wang Z, Liang Y, Guan L, et al Genome wide association study and genomic prediction for fatty acid composition in Chinese Simmental beef cattle using high density SNP array. BMC Genomics. 2017; 18 (1): 464 10.1186/s12864-017-3847-7 28615065 PMC5471809

[126] Fortes MR, Kemper K, Sasazaki S, Reverter A, Pryce JE, Barendse W, et al Evidence for pleiotropism and recent selection in the PLAG1 region in Australian Beef cattle. Anim Genet. 2013; 44(6): 636–47. 10.1111/age.12075 23909810

[127] Bansal SK, Gupta N, Sankhwar SN, Rajender S. Differential genes expression between fertile and infertile spermatozoa revealed by transcriptome analysis. Plos One. 2015; 10 (5): e0127007 10.1371/journal.pone.0127007 25973848 PMC4431685

[128] Asadollahpour NH, Ayatollahi MA, Esmailizadeh A. Whole‐genome sequence analysis reveals candidate genomic footprints and genes associated with reproductive traits in Thoroughbred horse. Reproduction in Domestic Animals. 2020; 55(2): 200–208. 10.1111/rda.13608 31858623

[129] Adjaye J, Herwig R, Brink TC, Herrmann D, Greber B, Sudheer S, et al Conserved molecular portraits of bovine and human blastocysts as a consequence of the transition from maternal to embryonic control of gene expression. Physiol Genomics. 2007; 31 (2): 315–27. 10.1152/physiolgenomics.00041.2007 17595343

[130] Venhoranta H, Pausch H, Flisikowski K, Wurmser C, Taponen J, Rautala H. In frame exon skipping in UBE3B is associated with developmental disorders and increased mortality in cattle. BMC Genomics. 2014; 15 (1): 890 10.1186/1471-2164-15-890 25306138 PMC4203880

[131] Hiltpold M, Niu G, Kadri NK, Crysnanto D, Fang Z-H, Spengeler M, et al Activation of cryptic splicing in bovine WDR19 is associated with reduced semen quality and male fertility. 2020 10.1371/journal.pgen.1008804 32407316 PMC7252675

[132] Dolebo AT, Khayatzadeh N, Melesse A, Wragg D, Rekik M, Haile A, et al Genome-wide scans identify known and novel regions associated with prolificacy and reproduction traits in a sub-Saharan African indigenous sheep (Ovis aries). Mamm Genome. 2019; 30 (11–12): 339–352. 10.1007/s00335-019-09820-5 31758253 PMC6884434

[133] Marques DBD, Bastiaansen JWM, Broekhuijse MLWJ, Lopes MS, Knol EF, Harlizius B, et al Weighted single-step GWAS and gene network analysis reveal new candidate genes for semen traits in pigs. Genet Sel Evol. 2018; 50 (1): 40 10.1186/s12711-018-0412-z 30081822 PMC6080523

[134] Cochran SD, Cole JB, Null DJ, Hansen PJ. Single nucleotide polymorphisms in candidate genes associated with fertilizing ability of sperm and subsequent embryonic development in cattle. Biol Reprod. 2013; 89 (3): 69 10.1095/biolreprod.113.111260 23904513

[135] Song H, Zhu L, Li Y, Ma C, Guan K, Xia X, et al Exploiting RNA-sequencing data from the porcine testes to identify the key genes involved in spermatogenesis in Large White pigs. Gene. 2015; 573 (2): 303–309. 10.1016/j.gene.2015.07.057 26192463

[136] Kasimanickam RK, Kasimanickam VR, Arangasamy A, Kastelic JP. Sperm and seminal plasma proteomics of high- versus low-fertility Holstein bulls. Theriogenology. 2019; 126: 41–48. 10.1016/j.theriogenology.2018.11.032 30529997

[137] Mezera MA, Li W, Edwards AJ, Koch DJ, Beard AD, Wiltbank MC. Identification of stable genes in the corpus luteum of lactating Holstein cows in pregnancy and luteolysis: Implications for selection of reverse-transcription quantitative PCR reference genes. J Dairy Sci. 2020; 103 (5): 4846–4857. 10.3168/jds.2019-17526 32229123

[138] Cesar AS, Regitano LC, Poleti MD, Andrade SC, Tizioto PC, Oliveira PS, et al Differences in the skeletal muscle transcriptome profile associated with extreme values of fatty acids content. BMC Genomics. 2016; 17 (1): 961 10.1186/s12864-016-3306-x 27875996 PMC5120530

[139] Jia H, Li X, Liu G, Loor JJ, Bucktrout R, Sun X, et al Perilipin 5 promotes hepatic steatosis in dairy cows through increasing lipid synthesis and decreasing very low density lipoprotein assembly. J Dairy Sci. 2019; 102 (1): 833–845. 10.3168/jds.2018-15208 30415861

[140] Ha N-T, Drögemüller C, Reimer C, Schmitz-Hsu F, Bruckmaier RM, Simianer H, et al Liver transcriptome analysis reveals important factors involved in the metabolic adaptation of the transition cow. Journal of Dairy Science. 2017; 100 (11): 9311–9323. 10.3168/jds.2016-12454 28865861

[141] Xu L, Yang L, Zhu B, Zhang W, Wang Z, Chen Y, et al Genome-wide scan reveals genetic divergence and diverse adaptive selection in Chinese local cattle. BMC Genomics. 2019; 20 (1): 494 10.1186/s12864-019-5822-y 31200634 PMC6570941

[142] Roh SG, Kuno M, Hishikawa D, Hong YH, Katoh K, Obara Y, et al Identification of differentially expressed transcripts in bovine rumen and abomasum using a differential display method. J Anim Sci. 2007; 85 (2): 395–403. 10.2527/jas.2006-234 17235024

[143] Hayes BJ, Pryce J, Chamberlain AJ, Bowman PJ, Goddard ME. Genetic architecture of complex traits and accuracy of genomic prediction: coat colour, milk-fat percentage, and type in Holstein cattle as contrasting model traits. Plos Genet. 2010; 6 (9): e1001139 10.1371/journal.pgen.1001139 20927186 PMC2944788

[144] Qanbari S, Pausch H, Jansen S, Somel M, Strom TM, Frieset R, et al Classic selective sweeps revealed by massive sequencing in cattle. Plos Genet. 2014; 10 (2): e1004148 10.1371/journal.pgen.1004148 24586189 PMC3937232

[145] Mészáros G, Petautschnig E, Schwarzenbacher H, Sölkner J. Genomic regions influencing coat color saturation and facial markings in Fleckvieh cattle. Animal genetics. 2015; 46 (1): 65–68. 10.1111/age.12249 25515556

