# Supplementary figures and images for: Selection signatures in two oldest Russian native cattle breeds revealed using high-density single nucleotide polymorphism analysis
Source: PLoS One. 2020 Nov 16;15(11):e0242200. doi: 10.1371/journal.pone.0242200 (PMC7668599; doi:10.1371/journal.pone.0242200)

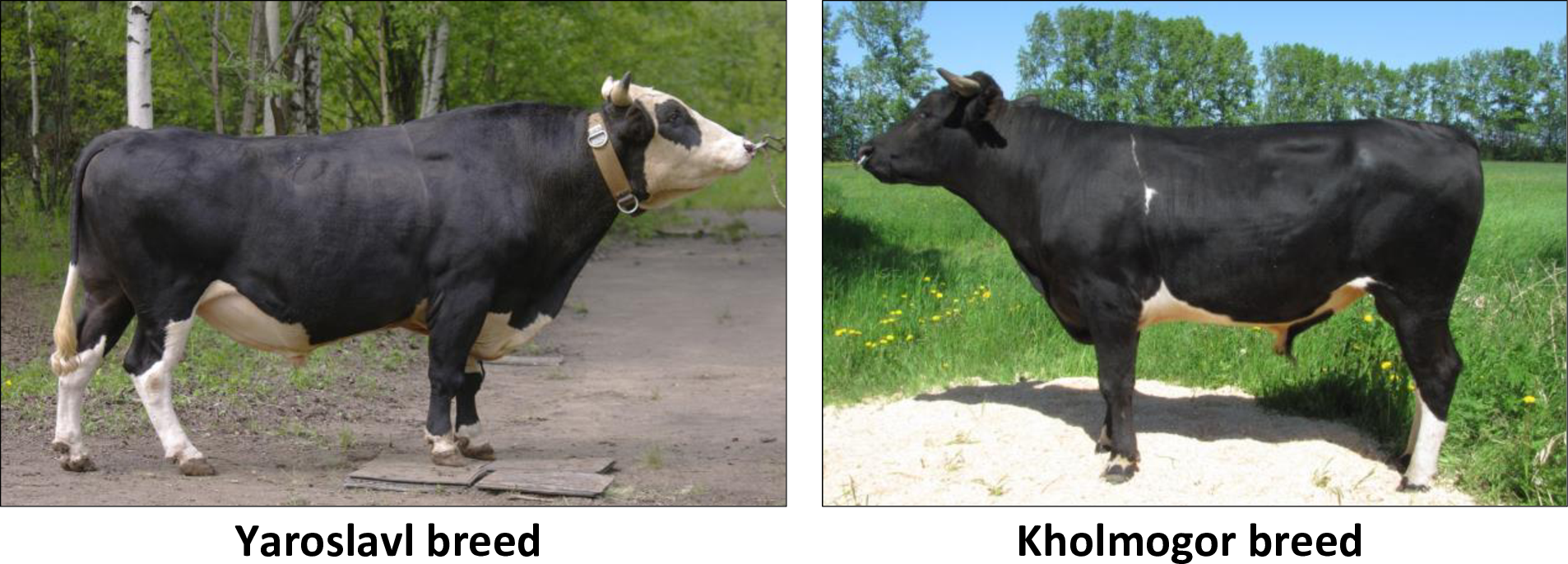

Supplement: S1 Fig — (TIF) [file pone.0242200.s001.tif]

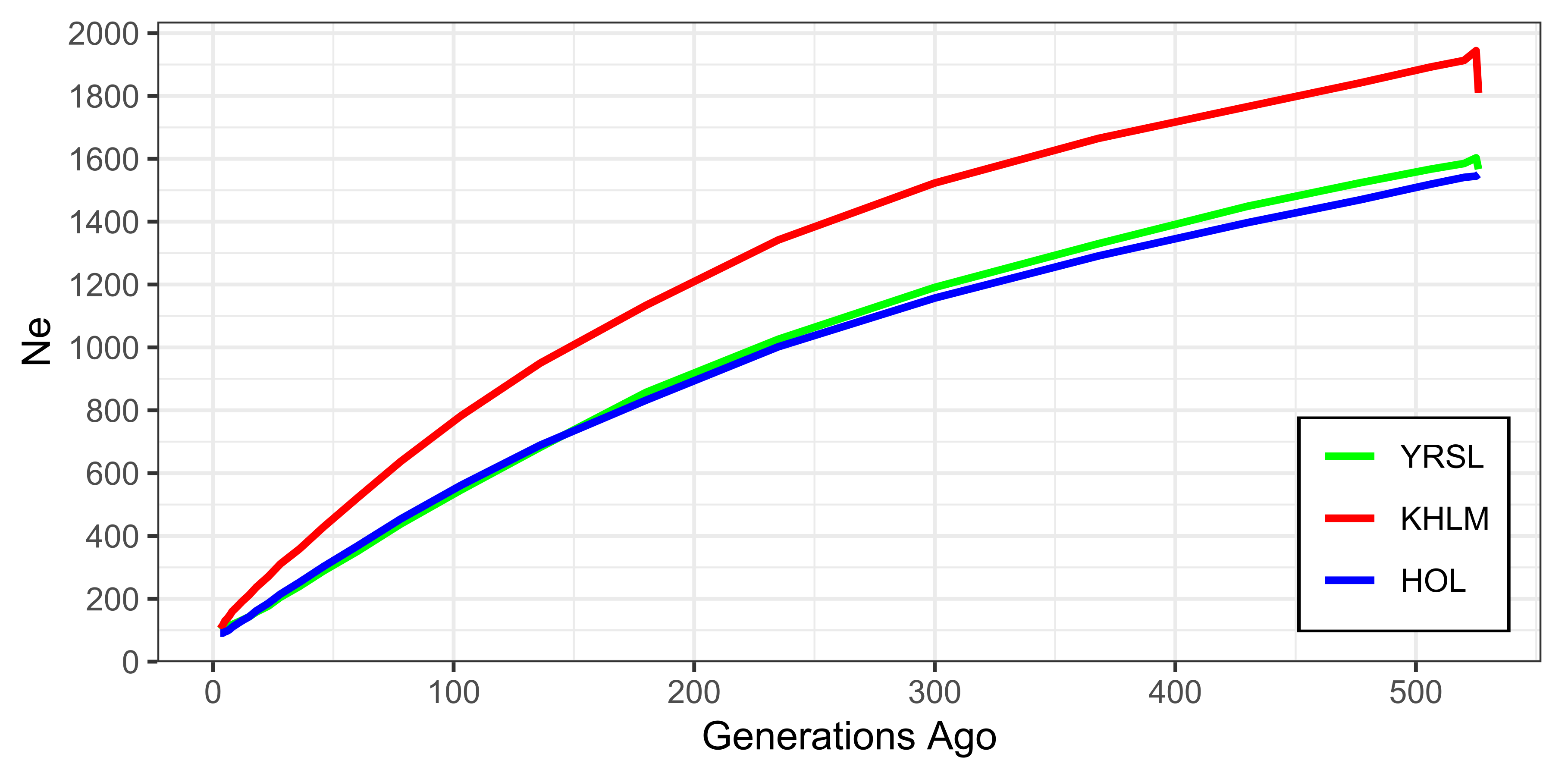

Supplement: S2 Fig — Note: Breeds: YRSL, Yaroslavl; KHLM, Kholmogor; HOL, Holsteins. (TIF) [file pone.0242200.s002.tif]

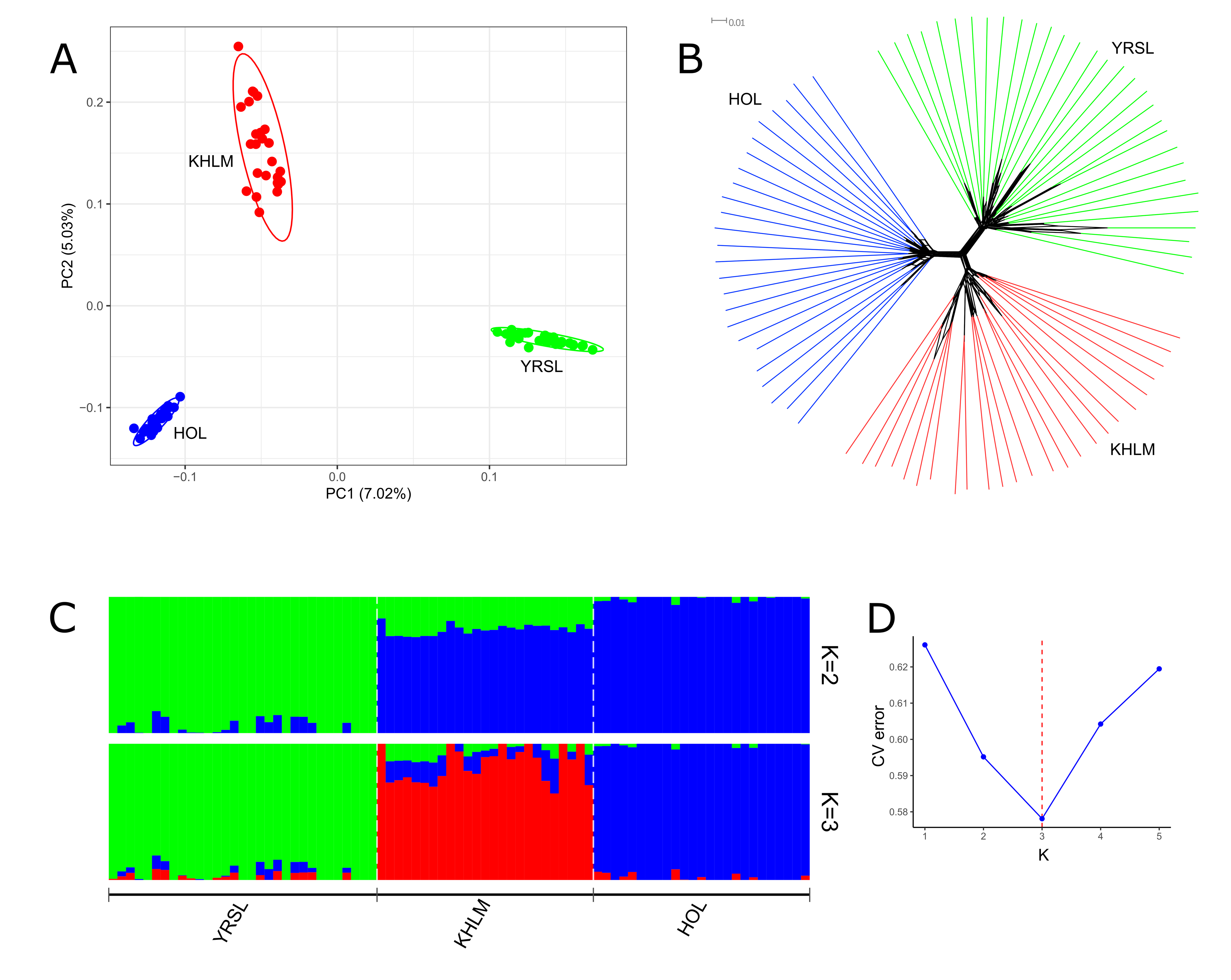

Supplement: S3 Fig — (A). Principal Component Analysis (PCA) plot showing the distribution of the studied cattle breeds in the dimensions of two coordinates. (B). A Neighbor-Net tree constructed on the basis of pairwise nucleotide genetic distances among individuals of the three studied breeds. (C). Admixture plot represents the cluster structure of the studied breeds for the number of clusters (K) 2 and 3. Different colours correspond to different ancestral populations. (D). Cross-validation (CV) error for the different number of clusters. Note: Axis X, first principal component (PC1); Axis Y, second principal component (PC2). The part of total genetic variability, which can be explained by each of the two components are indicated in curly parentheses. Breed: YRSL, Yaroslavl; KHLM, Kholmogor; HOL, Holsteins. (TIF) [file pone.0242200.s003.tif]

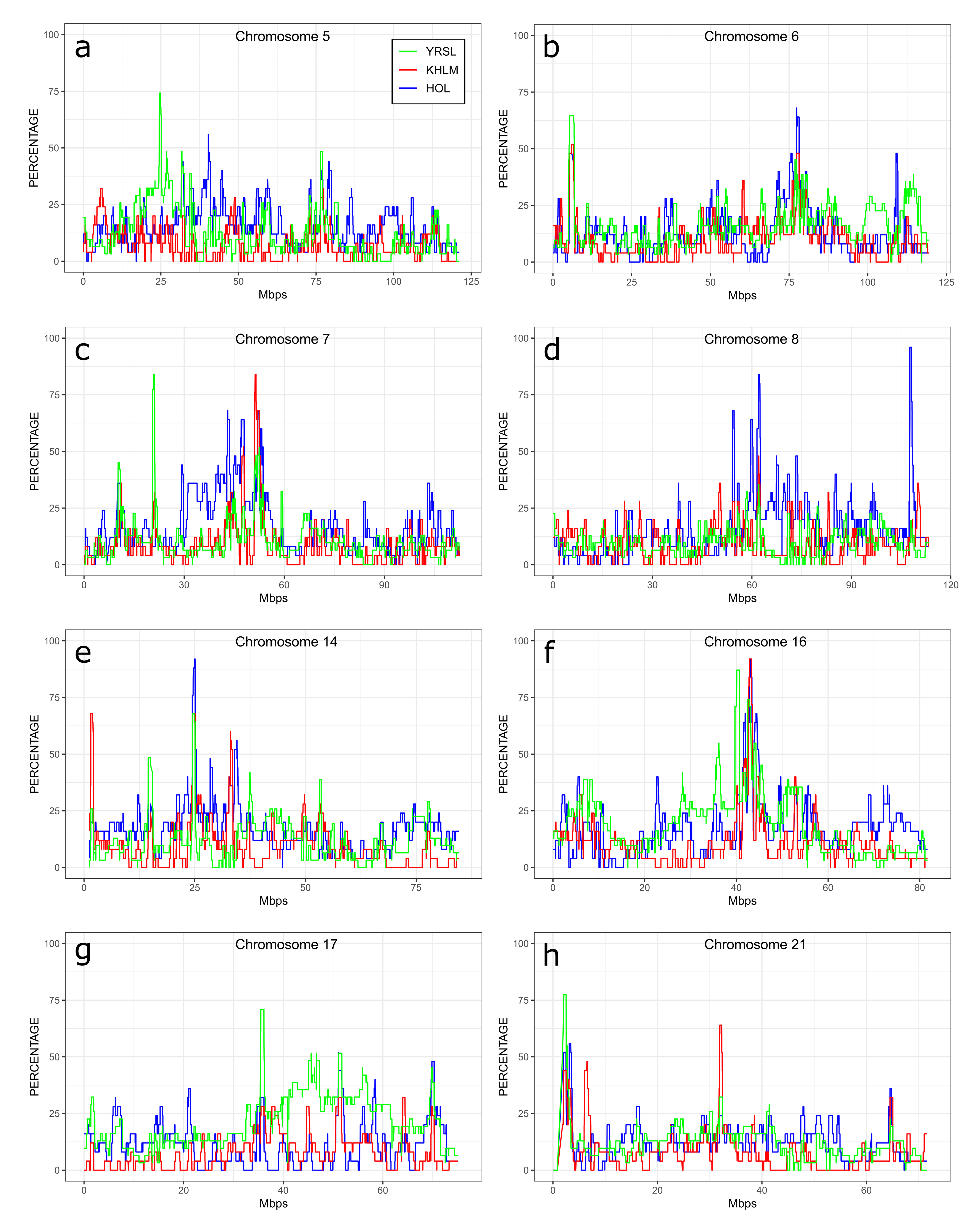

Supplement: S4 Fig — (a). BTA5, (b). BTA6, (c). BTA7, (d). BTA8, (e). BTA14, (f). BTA16, (g). BTA17, (h). BTA21. Note: Axis X, chromosomal positions (Mbps); Axis Y, the ratio of animals carrying ROHs (in percent); YRSL, Yaroslavl; KHLM, Kholmogor; HOL, Holsteins. (TIF) [file pone.0242200.s004.tif]
