# Supplementary material for: Selection signatures in two oldest Russian native cattle breeds revealed using high-density single nucleotide polymorphism analysis
Source: PLoS One. 2020 Nov 16;15(11):e0242200. doi: 10.1371/journal.pone.0242200 (PMC7668599; doi:10.1371/journal.pone.0242200)
Supplement: Attachment — Submitted filename: Response to Reviewers.doc [file pone.0242200.s013.doc]

Dear Ms Gyaneshwer Chaubey,

We are grateful for your consideration of this manuscript and for opportunity to submit the revised version of the PONE-D-20-15794 «Selection signatures in two oldest Russian native cattle breeds revealed using high-density single nucleotide polymorphism analysis» by Natalia Zinovieva et al. to PLOS ONE.

We would like to express our sincere gratitude to the Reviewers for the attention to our paper and for all valuable comments and suggestions, which helped us to improve the quality of the manuscript. We agreed with all comments and our responses are indicated below in a point-to-point manner.

We deposited our Dataset files in the Dryad Digital Repository. Zinovieva, Natalia et al. (2020), Selection signatures in two oldest Russian native cattle breeds revealed using high-density single nucleotide polymorphism analysis, Dryad, Dataset, <https://doi.org/10.5061/dryad.vt4b8gtq9>. The Dataset will be free available upon the manuscript will be accessed for publication. The editors and reviewers could access the Dataset files using following URL: <https://datadryad.org/stash/share/UDZHQNBg-m05QtB3jtPLze6UTRRXMXd5xGUmBDhjF3I>

We hope that our revised manuscript will facilitate the decision to publish our study in PLOS ONE. In any case, we are open to consideration of any further comment on our answers.

Sincerely yours,

Natalia Zinovieva

Biol. Sci., Professor

Member of the Russian Academy of Science

Director of the L.K. Ernst Institute of Animal Husbandry

[Phone number] +7(4967) 65-11-63

[Email address] [n_zinovieva@mail.ru](mailto:n_zinovieva@mail.ru)

**Response to the Editor**

In your Methods section, please state the volume of the blood samples collected for use in your study.

**Response:**

We indicated the volume of collected blood samples – 5 ml of whole blood (L143).

**Responses to the Reviewer №1:**

1. This manuscript present analysis of selection signature in two Russian breeds using bovine SNP chip panel. The analysis and results presented are detailed.
My main concern is on the very small samples size (31 and 25) used in the study presented. These samples may not be enough to represent the haplotypes present in these populations, hence may not be repeatable if another sample from these population are tested. Most of the estimates are based on underlying LD which will require large samples size for reliable estimates of LD. It should be established and discussed if the samples were enough.

**Response:**
We agreed with the Reviewer. Considering the relatively small sample size, we were very thoughtful when choosing animals for research. The selected individuals represented different sire lines and different breeding farms, and revealed the low amount of Holstein ancestral components (S3 Figure). We specified it in Materials and methods section (L152-L155) and discussed in the Discussion section (L511 - L515).

2. The manuscript is too long and will require restructuring. Most of the results (tables and graphs) related to the individual three tests can go into supplementary. The key selection signature obtained from combining the tests could be included. E.g. Table 2, Table 3, Table 4 can be moved to the supplementary. Table 5 (or 4) can stay in the main manuscript. The main manuscript can focus on the main novel regions identified. The length of the manuscript can be easily reduced to one third.

**Response:**

We restructured the manuscript according to the suggestions of the Reviewer. We moved the PCA, individual three and Admixture results (Fig. 3) as well as the distribution of the ROH islands (Fig. 7) to the Supplemental data (S3 Fig and S4 Fig. in the revised manuscript). We moved the Table 2 and 3 (S4 and S5 Tables in the revised manuscript, respectively) to the supplemental data.

We significantly shortened the discussion section, focusing on the most important observations. We described in details only the genomic regions, which were common for three breeds and provided only a brief analysis of other breed-specific regions. We tried to find the relationships between functions of identified candidate genes and the history of breeds. The length of the manuscript was reduced from 52 sites to 34 sites (excluding references).

3. Abstract could be more informative – including specific results: e.g. regions discovered.
**Response:**

We made the Abstract section more specific and identified genomic regions were pointed out.

4. Line 23: any cattle breed may carry selection signature – why Russian native only. – may rephrase.

**Response:**
We rephrased the sentence.

5. Line 28: “in which most of the ancestral genomic components are retained. – how this was established?

**Response:**
In our previous study of nine Russian cattle breeds (Sermyagin A.A. et al., *GSE*, 2018) based on the medium-density SNP BeadChips we identified the group of Russian cattle breeds including the Yaroslavl and Kholmogor breeds, which have been the least influenced by introgression with non-Russian breeds. In our last study of the historical specimens of Kholmogor and Yaroslavl cattle, dated from the end of 19^th^ to the first half of 20^th^ century using microsatellites (Abdelmanova A.S. et al., *Genes*, 2020) we confirmed the maintenance of historical components in modern populations of above-mentioned breeds. We added the paper of Abdelmanova et. al. to the references (ref. 11). Considering that these results belong to our previous studies, we rephrased the sentence in the Abstract section and made additional explanation in the Introduction section (L80 - L83).

6. Line: 46: “genomic regions and candidate genes that might be under putative selection” – I guess it should be putative regions and candidate genes.
**Response:**
We corrected the sentence.

7. ROH may be because of random drift or recent inbreeding and may not be specific to the selection signatures. –
**Response:**
We agree with the Reviewer. Considering that genetic drift or recent inbreeding may have an impact on ROHs (Kim ES et al., 2013, ref. 65), we selected the ROH islands to be under selection pressure, if they were distributed in more than 50% of animals and were confirmed by at least two methods or if the identified ROH islands were observed in more than 70% of animals. We added this information to the Discussion section (L575 – 578).

8. Line 254: these signature could be compared with the meta-assembly of selection signature in cattle (https://doi.org/10.1371/journal.pone.0153013 )
**Response:**
We performed the comparison of the identified genomic regions with the meta-assembly of selection signature in cattle. We summarized the results in supplementary (S7 Table) and discussed it in the main text of the Manuscript (L577 – L578, L591- L594)

9. Line 309: should be “Estimates of ROHs and Froh”

**Response:**

We corrected the title of the paragraph.

**Responses to the Reviewer №2:**

This is a manuscript in which the authors prepared a small but adequate amount of genotypic data on two Russian breeds of cattle to look for signatures of selection. Similar works (citations 31, 32) have been published before on a wider range of Russian breeds, but the tool used in those studies had a sever-fold lower density of SNP.

**Response:**

Thank you very much for the positive evaluation of our manuscript.

1. The main reason to study rare or dying breeds is to try and find genetic variation that is useful to understand the biology of cattle for use in future generations as humanity moves to a more monoculture approach to livestock farming. It would be very much appreciated if the authors would move the historic information and description of the breed type (dairy or beef or dual-purpose) to the introduction of the article. This provides the entire justification as to why the work has value and if the experiment will yield results of interest. Otherwise, one has to go to google to see and find out about these breeds, one which looks like an old time Holstein and the other looks like a black-white face dairy animal (both horned).
**Response:**

We expanded the description of the studied cattle breeds in the Introduction section by specifying the breed type (dairy) and adding the relevant historical information (L60 – L64, L67 – L71, L73 – L76, L80 – L83). We added a citation of our recent study of the historical specimens of Kholmogor and Yaroslavl cattle, dated from the end of 19^th^ to the first half of 20^th^ century using microsatellites (Abdelmanova et al., genes, 2020, ref. 11), confirming their earlier differentiation from Holstein ancestors.

2. In the introduction - Lines 93-109 are not necessary. Be succinct and summarize in a few sentences why the 3 method approach is better.
**Response:**

We shortened the paragraph (L115 - L121)

3. In the methods - its not quite clear. Were genes nearby but outside the zones of selection still considered as candidates. Genes beyond the detectable boundaries of the selection signatures should not be in the positional candidate genes list.
**Response:**

We sincerely appreciate this valuable comment. The windows of 0.4 Mb (0.2 Mb upstream and 0.2 Mb downstream of the top 0.1 % SNPs) were selected only for SNPs, which were identified by *F*_ST_ statistics. We provided the full list of genes, which were entirely or partly localized within selected windows (see S2 Table) with the aim to compare the genes with those, which were identified by hapFLK and ROH analysis. We consider the genes as positional candidate genes only if the causal SNPs were localized within genes. These genes were marked in bold in S2 Table. For hapFLK and ROH analyses, the genes were selected only if they were localized entirely or in part within the identified genomic regions. Table 4 includes only the genes, if they were localized within genomic regions identified by hapFLK and ROH analysis. The results of *F*_ST_ statistics were used only if they were overlapped with hapFLK and ROH analysis. In Table 4, we marked by asterisks the genes, which were localized within genomic region identified by hapFLK and ROH analysis, and thus could be considered as positional candidate genes and albeit were not overlapped with causal SNPs, selected by *F*_ST_ analysis, but were localized within 0,4 Mb windows of such SNPs. We have carefully checked the entire text and removed any inaccuracies in the use of the term positional candidate genes.

4. Population size - suggest moving lines 293-295 into the subsequent section where it belongs.

**Response:**

In accordance with the Reviewer’s suggestion, we moved the sentence concerning Ne in the subsequent section (L307 – L308).

5. The authors were given HD data from a set of Holsteins cited as Bahbahani et al.; however, this citation is not appropriate since this data was contributed to that paper as well. The original source of the Holstein data should be cited, as this is important to provide context as to what animals are in this cohort and how they were selected. If they are the animals from the Hapmap, then they would represent a diverse set of the most highly used AI bulls in USA history. Not really a reflection of European Holsteins or even Holsteins from Holland (at least 5 generations removed).

**Response:**

We checked thoroughly the paper of Bahbahani et al. (2017). According to the Materials and Methods section, the authors independently conducted genotyping of Holsteins using HD BeadChips. The row genotypes were deposited in Dryad Digital Repository as SNP genotypes data from Illumina BovineHD Genotyping BeadChip of the examined cattle populations under URL <https://doi.org/10.5061/dryad.38jp6>.

The entire sample of Holstein breed, genotyped by Bahbahani et al. included 63 animals of both sexes (bulls and cows). We performed the kinship analysis and avoided the close-related animals (parent-offspring and full- or half-sibling) and selected for our study 25 non-related individuals of Holstein breed of both sexes.

We added in the Manuscript the reference on the source of SNP data for Holsteins (ref. 35)

6. Were the marker heterozygosity calculations for Holsteins comparable to previously published works (i.e. Hapmap)?
**Response:**

We added in the Discussion section (L533 – L536) the comparison of inbreeding coefficient, detected in our study, with the one which were found in other recent reports, including HapMap (Huson HJ et al., ref. 61).

7. The results are full of a lot of lists to try and comprehensively describe all the results. Comparing highest and lowest chromosomes really doesnt matter in the big picture. What matters are the regions in common between breeds. At this point in the article - one is wondering why they would have regions in common with Holstein, since we dont know if the Russian breeds are dairy or beef animals.

**Response:**

According to the Reviewer’s suggestion, we expanded the description of studied breeds in the Introduction section, specifying the breed type (dairy) and short history of breeds (L60 – L64, L67 – L71, L73 – L76, L80 – L83). We added a citation of our recent study of the historical specimens of Kholmogor and Yaroslavl cattle, dated from the end of 19^th^ to the first half of 20^th^ century using microsatellites (Abdelmanova et al., genes, 2020, ref. 11), confirming their earlier differentiation from Holstein ancestors.

We rewrote and significantly shortened the Discussion section. We provided the detail description of two genomic regions on BTA14 and BTA16, which are common for three breeds (Yaroslavl, Kholmogor and Holsteins). The other identified breed-specific genomic regions were only briefly described. We tried to find the possible explanations of relationships between functions of the identified candidate genes and the history of the studied breeds and provided these in the Discussion section.

8. It would have been more intriguing to see another continental (Fleckvieh/Hereford) or island breed and also a Mongolian or N. Chinese breed of cattle and maybe a zebu breed for contrast in the PCA.
**Response:**

Thank you for your suggestion. In our previous study of nine Russian cattle breeds, using medium-density SNP BeadChip (Sermyagin A.A. et al., GSE, 2018, <https://gsejournal.biomedcentral.com/track/pdf/10.1186/s12711-018-0408-8>) we have considered the Russian cattle breeds in context of European and worldwide breeds. Because the high-density SNP genotypes are still available for a limited number of breeds, we did not consider the studied breeds in comparison with the world breeds.

9. Is the result of nearly equal number of Fst SNPs between all 3 breeds surprising - considering the Holstein are under more intense selective pressure than the Russian breeds? The other 2 methods suggest this hypothesis is true (more ROH and FLKhap in Holsteins.

**Response:**

The top 0.1% SNP for *F*_ST_ values were considered to be under selection pressure. The differences between pairs of breeds were low, because the number of polymorphic SNPs, which were selected for the analysis for the studied pairs of breeds, was similar.

10. The SNP in the intron of PLAG1 still has not been proven as the causative mutation, its purely speculative. There is another paper (Yuri Tani et al.) that disputes this with very strong evidence. This should be toned down in citation.
**Response:**

We thoroughly analyzed the original papers describing the effect of PLAG1 genes on stature. We found an additional recently published report confirming the effect of causative SNP on stature in Chinese cattle breeds (Hou et al., Animal Genetics, 2020). We included this citation in the Manuscript (ref. 83). Unfortunately, we did not find another paper of Yuri Tani Utsunomiya et al. concerning PLAG1. Could the Reviewer, please, provide us with the reference? According to the Reviewer’s suggestion, we rewrote this part of the Discussion section and toned down the description of possible effect of PLAG1 in relationship with stature characteristics of studied breeds (L612 – L617).

11. Overall, a nice piece of thesis work; but the paper should not be presented like a thesis. The discussion is way too long, and should be trimmed down to highlight only the most important findings. The paper should be halved in length. The PCA and admixture should be moved to supplemental data, since they dont give very much insight (only 3 breeds in comparison has its drawbacks for interpretation).

**Response:**

Thank you very much for positive evaluation of our manuscript. We tried to take into account all the comments and made necessary corrections to the text of the manuscript.

We significantly shortened the discussion section, highlighting the most important observations. We described in details only the genomic regions, which were common for three breeds and provided only a brief analysis of other breed-specific regions. We tried to find the relationships between gene functions of identified candidate genes and the history of breeds. We moved the PCA and Admixture results (Fig. 3), as well as the Distribution of ROH islands (Fig. 7) to the Supplemental data (Fig. S3 and S4 in the revised manuscript, respectively). According to the suggestion of the Reviewer 1, we moved to the supplemental data the Table 2 and 3 (S4 and S5 Tables in the revised manuscript, respectively).
